# Characterization of Rat Cardiovascular System by Anacrotic/Dicrotic Notches in the Condition of Increase/Decrease of NO Bioavailability

**DOI:** 10.3390/ijms21186685

**Published:** 2020-09-12

**Authors:** Lenka Tomasova, Anton Misak, Lucia Kurakova, Marian Grman, Karol Ondrias

**Affiliations:** 1 Institute of Clinical and Translational Research, Biomedical Research Center, Slovak Academy of Sciences, Dubravska Cesta 9, 845 05 Bratislava, Slovakia; lenka.tomasova@savba.sk (L.T.); anton.misak@savba.sk (A.M.); kurakova4@uniba.sk (L.K.); marian.grman@savba.sk (M.G.); 2 Department of Pharmacology and Toxicology, Faculty of Pharmacy, Comenius University, 832 32 Bratislava, Slovakia

**Keywords:** arterial pulse waveform, anacrotic notch, dicrotic notch, blood pressure, hemodynamic parameter, signaling pathway, nitric oxide, l-NAME

## Abstract

We characterized modes of action of NO-donor S-nitrosoglutathione (GSNO) and NO-synthase inhibitor l-NAME derived from dicrotic (DiN) and anacrotic (AnN) notches of rat arterial pulse waveform (APW) in the condition of increased/decreased NO bioavailability. The cross-relationship patterns of DiN and AnN with 34 hemodynamic parameters (HPs) induced by GSNO and l-NAME are presented. After GSNO bolus administration, approximate non-hysteresis relationships were observed in the difference between DiN–AnN (mmHg) blood pressure (BP) and other 19 HPs, suggesting that these HPs, i.e., their signaling pathways, responding to NO concentration, are directly connected. Hysteresis relationships were observed between DiN-AnN (mmHg) and other 14 HPs, suggesting that signaling pathways of these HPs are indirectly connected. The hysteresis relationships were only observed between the time interval DiN-AnN (ms) and other 34 HPs, indicating no direct connection of signaling pathways. The cross-relationship patterns of DiN-AnN (mmHg), but not DiN-AnN (ms), induced by l-NAME were in accordance to the increased NO bioavailability induced by GSNO. In conclusion, we found the non-hysteresis/hysteresis cross-relationship “patterns” of DiN-AnN intervals to other HPs in the presence of GSNO that revealed their direct or indirect signaling pathways connections. This may contribute to our understanding of biological effects of natural substances that modulate NO production and/or NO signaling pathways.

## 1. Introduction

Nitric oxide (NO) is involved in the modulation of numerous biological processes, for example, blood flow regulation, vascular tone, and platelet aggregation, and thus plays a crucial role in the development of cardiovascular diseases, such as hypertension, heart failure, ischemia, or stroke [1,2,3]. Therefore, NO and its signaling pathways are studied as molecular targets for therapeutic strategies for cardiovascular pathologies [3]. Several natural compounds, e.g., polyphenols, were suggested to modulate endogenous NO production by NO-synthases (NOS) or its signaling pathway, thus influencing cardiovascular homeostasis [1,2,4,5]. However, to introduce such natural agents in clinical practice, it is necessary to understand their complex interactions with biological objects in particular cardiovascular conditions [1,5]. NO-donors and NO-synthase inhibitors are useful in studies of NO actions in the cardiovascular system. S-nitrosoglutathione (GSNO) is suggested as a storage form of NO in vivo [6,7], and S-nitrosoglutathione reductase modulates NO availability in the cell by catalyzing the breakdown of GSNO [3]. N(ω)-Nitro-l-arginine methyl ester (l-NAME) is a widely used inhibitor of NOS, and the treatment with l-NAME induces hypertension resulting from decreased NO bioavailability [8]. Our study is focused on the characterization of the biological actions of GSNO and l-NAME in the cardiovascular system by detailed analysis of their effects on anacrotic (AnN) and dicrotic (DiN) notches of arterial pulse waveform (APW).

The information obtained from the shape of APW analysis can provide insight into the patho-physiological conditions of cardiovascular system. These data can contribute to the characterization of the specific modes of action of bioactive substances. However, only a few hemodynamic parameters (HPs) of APW in different patho-physiological conditions have been characterized so far [9,10,11,12,13,14,15,16,17,18,19,20,21]. To our knowledge, except for our two recent studies [22,23], there was neither a detailed detection of anacrotic notch nor reports of the time dependence of the cross-relationships of HPs.

Our previous and recent works have been based on the hypothesis that it is possible to characterize the cardiovascular system in many patho-physiological conditions just from the detail shape of APW. To characterize the cardiovascular system, a database of unique patterns for various cardiovascular conditions should be created by exploring numerous “patterns” of HPs and their cross-relationships derived from APW. This can be achieved by the characterization of APW HPs for particular pathological cardiovascular conditions, e.g., coronary artery disease, hypertension, mitral valve prolapse, arrhythmias, or for different model conditions by using selective drugs that activate or inhibit specific receptors and signaling pathways. If at least one of the “patterns” of HPs is unique to a particular patho-physiological condition or pathway, then it would be hypothetically possible to deduce from any obtained HPs whether the specific condition occurred.

We have analyzed changes in the shape of APW by introducing 35 HPs and calculating cross-relationships between HPs in the condition of increased/decreased NO bioavailability [22,23]. The obtained cross-relationships between 35 HPs provided “patterns” for these particular cardiovascular conditions. It was observed that changes of 35 HPs and some of their cross-relationships in the condition of decreased NO bioavailability were well fitted by simple mathematical functions [23]. In the condition of a transient increase of NO bioavailability, non-hysteresis/hysteresis cross-relationships between systolic BP and HPs were observed, suggesting direct or indirect signaling pathways [22]. From the cross-relationships of 35 HPs, one can obtain theoretically 595 “mutual patterns” and an indication of direct or indirect connections between the signaling pathways. Therefore, in the present work we extended evaluation of APW for the cross-relationships of AnN and DiN to other 34 HPs.

**Figure 1:**
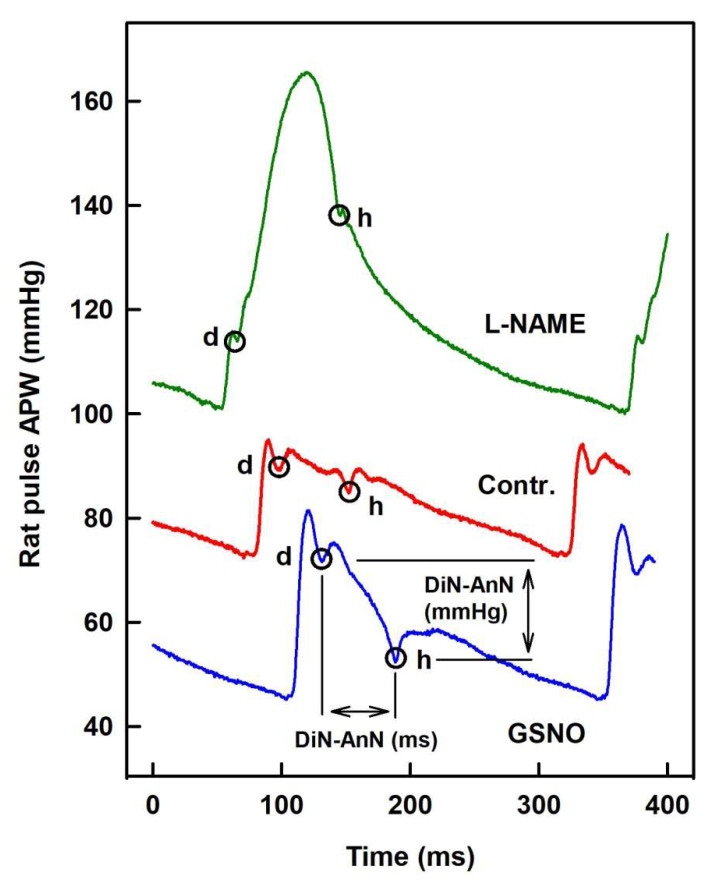
The left common carotid artery pulse waveform (APW) in the anesthetized rat. Anacrotic (AnN) notch (**d**) and dicrotic (DiN) notch (**h**) marked at control APW (red) and after i.v. administration of 32 nmol·kg^−1^ NO-donor S-nitrosoglutathione (GSNO) (blue) or 25 mg·kg^−1^ l-NAME (green). For the definition of time interval (DiN-AnN in ms) and the difference of blood pressure (BP) (DiN-AnN in mmHg) of dicrotic and anacrotic nothes, see Supplementary Materials Figure S1.

Two downward deflections can be observed on the APW, the AnN, and DiN (Figure 1). AnN appears at the ascending part of the systolic wave, shortly after the systolic peak. DiN appears at the time of the aortic valve closure and divides the APW into the systolic duration, from the onset of the APW to the DiN, and the diastolic duration, from the DiN to the onset of the next APW. Yet, the detailed origin of these deflections remains unclear. It was believed that DiN is caused by the aortic valve closure; however, recent studies suggest that DiN is created due to a backward pressure component caused by reflected waves at the periphery [24]. Importantly, changes in the time and position of DiN were observed after the modulation of NO bioavailability [14]. However, other vasoactive substances, e.g., propanolol, phentolamine, or phenylephrine, failed to influence the relative level of DiN [14,24], suggesting that the changes in the position of DiN are specific to NO signaling. In addition, a correlation between the position of DiN and the state of cardiovascular system was reported by several authors. For instance, decreased DiN time was observed during exercise and in athletes with high maximal oxygen uptake [25,26]. In contrast, increased DiN level and time has been reported in hypertensive and diabetic patients [16,17].

In our published experimental approach [22,23], AnN is clearly visible and its position is relatively easy to evaluate from APW (Figure 1). In the present work, using the high-resolution APW data [22], we exploited the clear AnN and DiN observation to characterize modes of action of GSNO and l-NAME in more detail. We found relationships of DiN and AnN with 34 hemodynamic parameters (HPs) derived from APW in a condition of transient increase/decrease of NO bioavailability induced by GSNO and l-NAME, respectively. From the cross-relationships of the notches with 34 HPs, the non-hysteresis/hysteresis “patterns” of the relationships were observed, from which their direct or indirect signaling pathways connections are suggested. The obtained time-dependent changes of DiN and AnN HPs, their time-dependent cross-relationships, and non-hysteresis/hysteresis connections provide patterns that characterize the cardiovascular conditions of increased/decreased NO bioavailability.

## 2. Results

### 2.1. The Cross-Relationships of 34 HPs to the Difference between BP of Dicrotic and Anacrotic Notches

**Figure 2:**
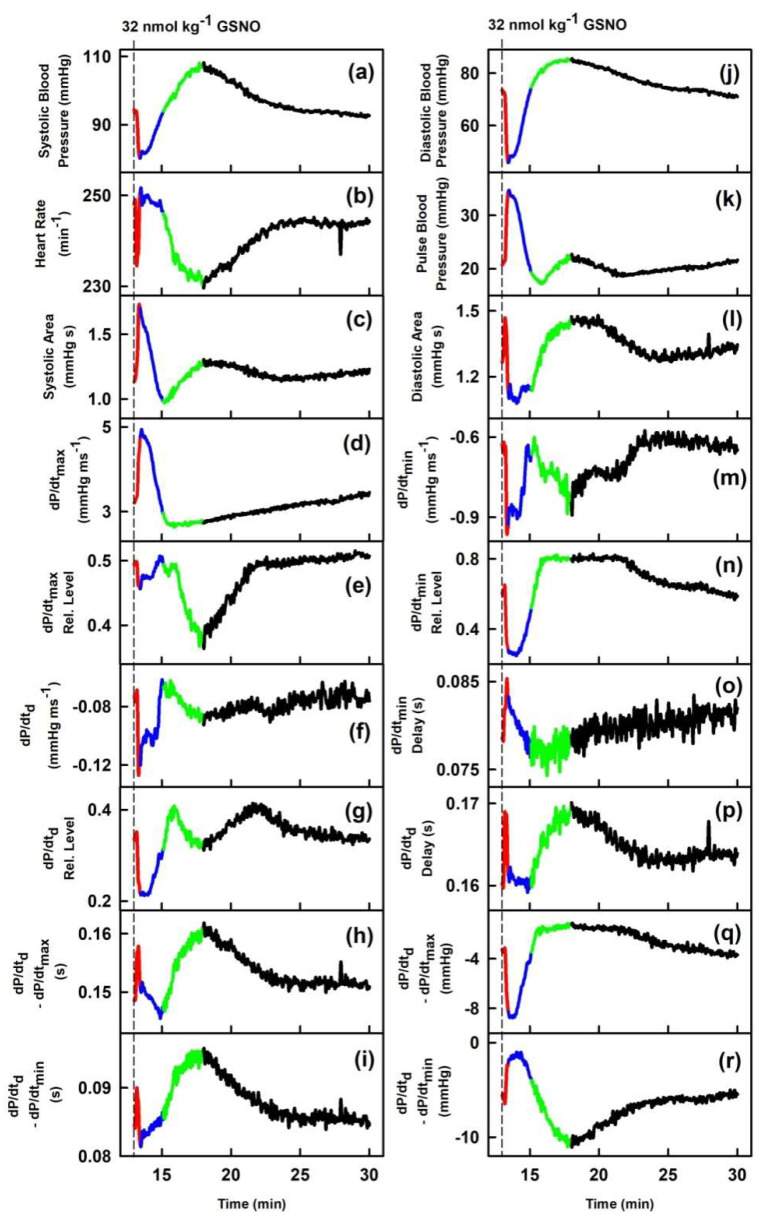

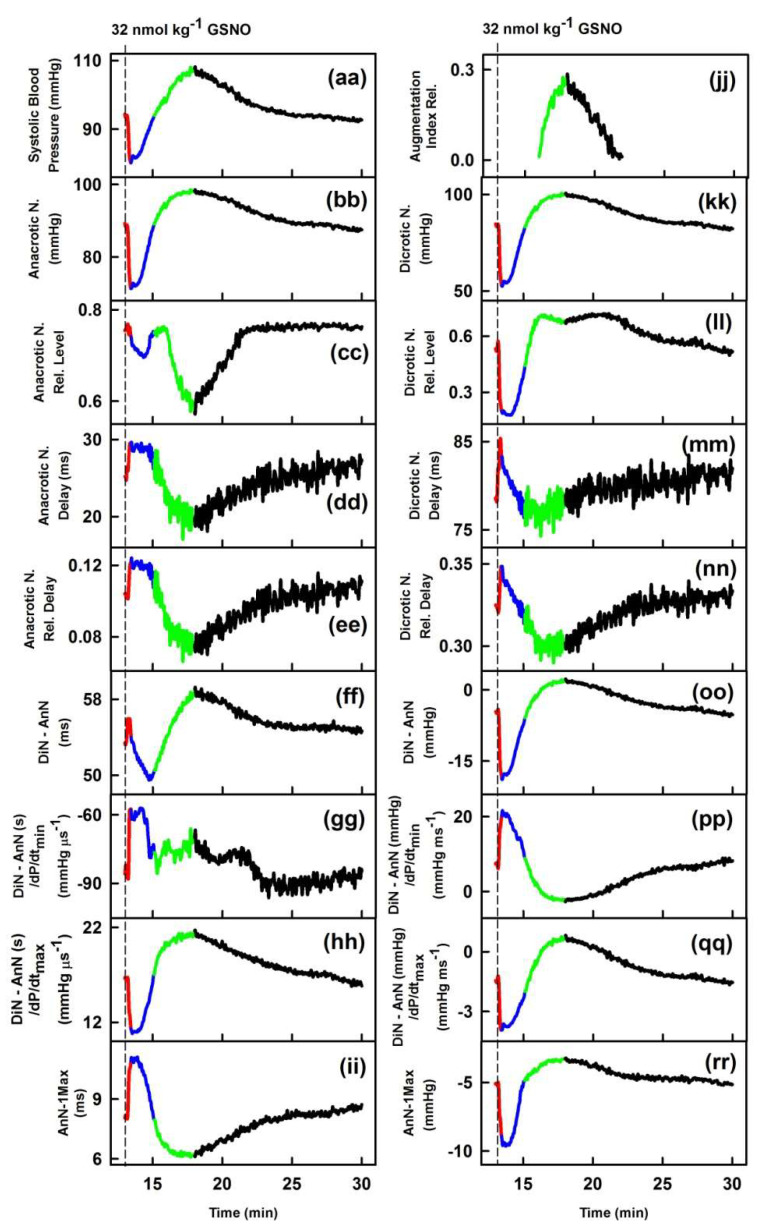
Time-dependent changes of hemodynamic parameters (HPs) of anesthetized rat after i.v. bolus administration of 32 nmol·kg^–1^ S-nitrosoglutathione (GSNO) (marked by dash lines). The red line starts 3 s before GSNO administration. Time period corresponding to the decrease of systolic BP (red), increase of systolic BP to the control value (blue), further increase of systolic BP to maximum (green), and decrease of systolic BP to the control value (black). Units in plots (**gg**), (**hh**), (**pp**), and (**qq**) are informative only.

We have previously characterized the cross-relationships of 34 HPs to systolic BP after GSNO administration [22]. In order to extend our previous work for other HPs, particularly to introduce the relationships to AnN and DiN, we have evaluated cross-relationships of 34 HPs to pressure and time interval differences of DiN-AnN from the data acquired in [22]. Figure 1 shows that AnN and DiN are clearly visible and their mmHg/ms positions depend noticeably on the condition of increased/decreased NO bioavailability. Figure 2 shows an example of time-dependent changes of 35 HPs after GSNO administration. The time dependency was divided to four phases: decrease of systolic BP (red), increase back to the control value (blue), further increase to maximum BP (green), and final decrease to the control value (black), in order to compare the time-dependent changes of particular segments between HPs. Some of the HPs followed similar or reverse time-dependent changes such as systolic BP (a). However, some HPs, e.g., heart rate (b), dP/dt_max_-RL (e), dP/dt_d_ (f), dP/dt_min_ (m), dP/dt_min_-delay (o), AnN-RL (cc), or DiN-AnN/dP/dt_min_ (gg), showed a completely different course in time, suggesting that the changes are activated at different time points and probably by different signaling pathways than the changes in systolic BP (Figure 2).

**Figure 3:**
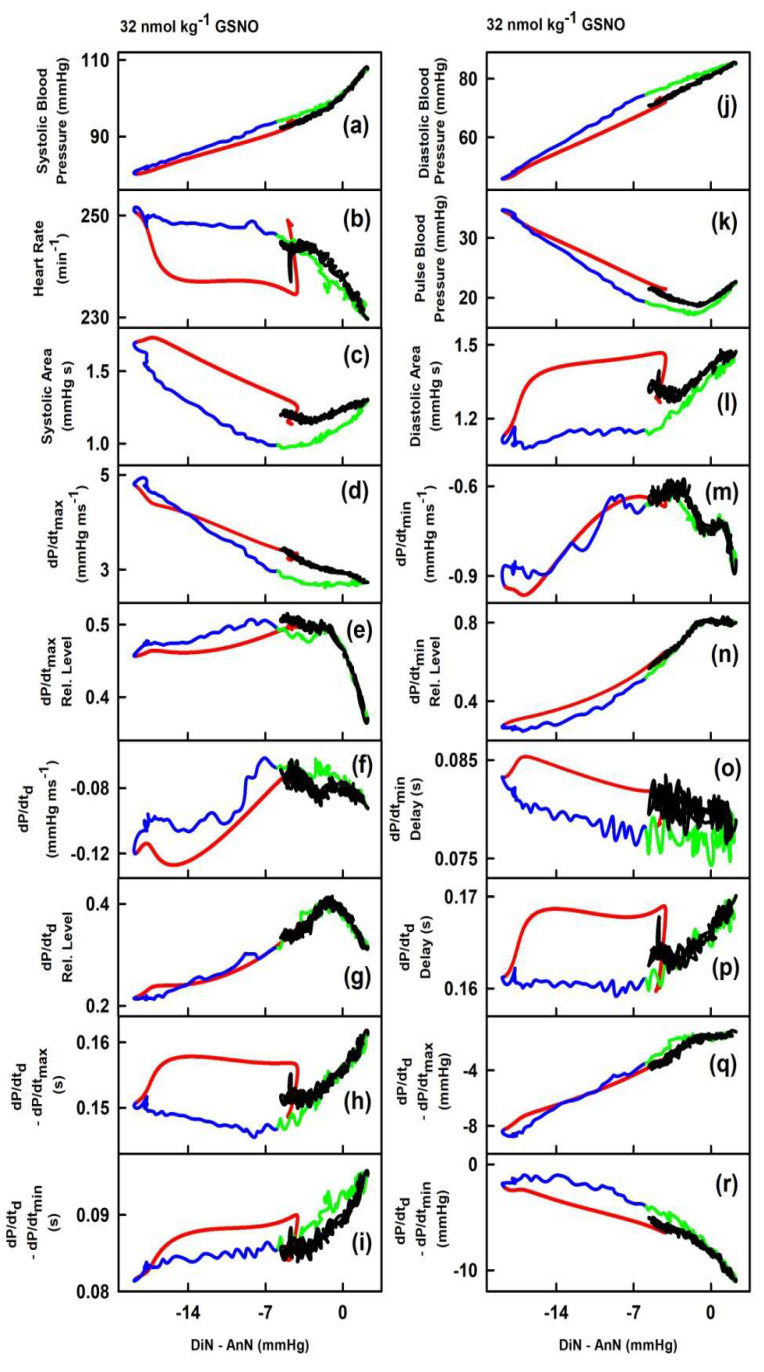

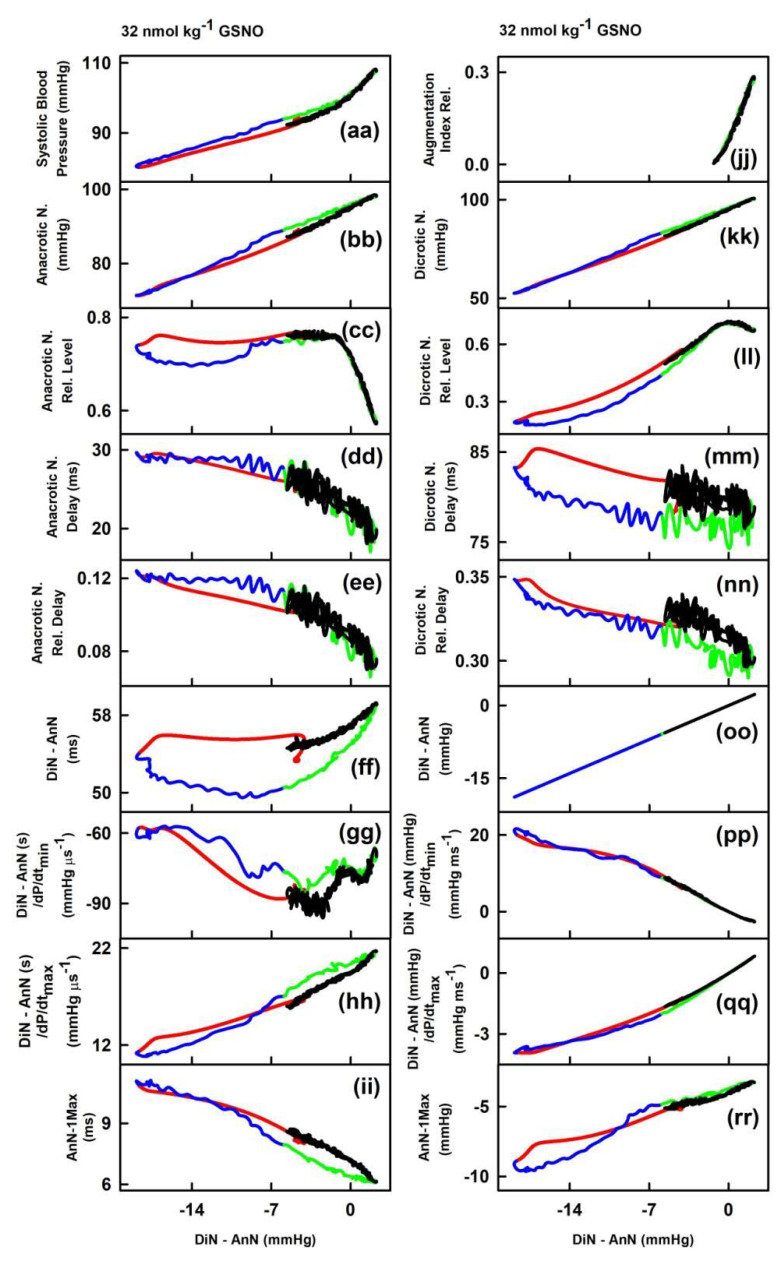
Two-dimensional relationships of HPs to the pressure interval between dicrotic (DiN) and anacrotic (AnN) notches after the administration of 32 nmol·kg^–1^ GSNO. The colors and time-dependent data correspond to Figure 2. Time period corresponding to the decrease of systolic BP (red), increase of systolic BP to the control value (blue), further increase of systolic BP to maximum (green), and decrease of systolic BP to the control value (black). The hysteresis was arbitrary defined as HPs-(DiN-AnN in mmHg) loop > 5 mmHg of DiN-AnN. The non-hysteresis was arbitrary defined as HPs-(DiN-AnN in mmHg) loop ≤ 5 mmHg of DiN-AnN.

In order to observe the two-dimensional cross-relationships of the 34 HPs to the difference between pressure of DiN and AnN (DiN-AnN in mmHg) after GSNO administration, the experimental data of the time-dependent changes of DiN-AnN in mmHg (Figure 2, plot (oo)) were used as x-axis values (Figure 3), and for other six rats in Figure S2. For the full description of the cross-relationships of the 34 HPs to the DiN-AnN (mmHg), each plot in Figure 3 should be three dimensional, which we found visually confusing. Therefore, we omitted time-dimension and present two-dimensional cross-relationships only. The time in the plots of Figure 3 starts at the point −4.8 mmHg and continues to the point −18.6 mmHg (red line), then continues to −6 mmHg (blue line), further to 2.1 mmHg (green line), and finally to the point −5.3 mmHg (black line).

Firstly, we evaluated the cross-relationships during decrease (red line) and increase (blue line) of systolic BP, whether the cross-relationship shows hysteresis or non-hysteresis.

**Figure 4:**
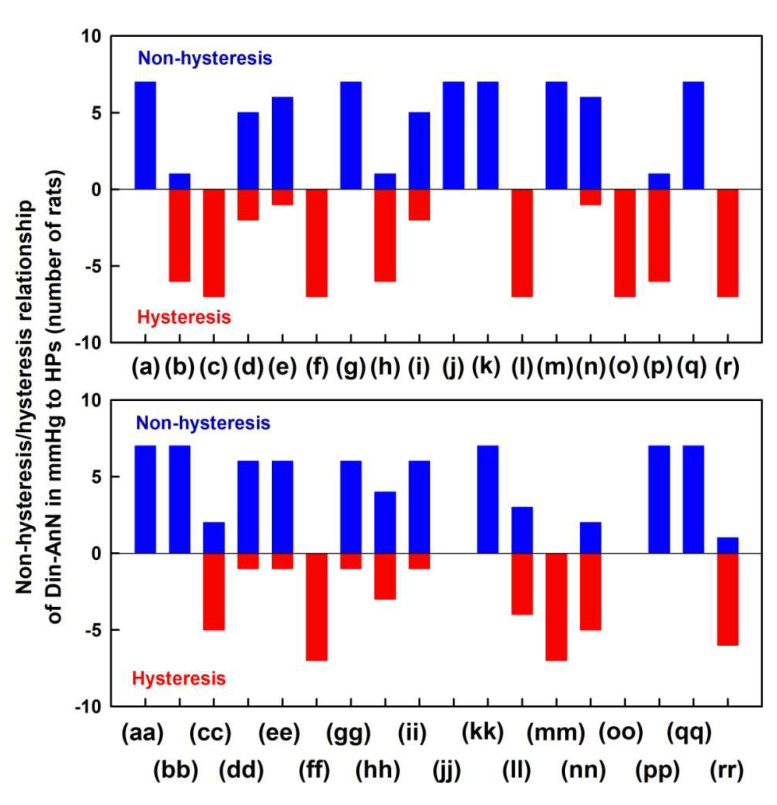
Non-hysteresis/hysteresis patterns of the two-dimensional relationships of HPs to pressure differences of DiN-AnN in mmHg. Data were taken from Figure 3 and Figure S2 (red and blue lines, which represent the time interval of decrease and increase of BP after administration of 32 nmol·kg^–1^ GSNO). Number of rats in which non-hysteresis (blue) or hysteresis (red) patterns were observed (*n* = 7). The hysteresis was arbitrarily defined as HPs-(DiN-AnN in mmHg) loop > 5 mmHg of DiN-AnN (in mmHg).

The non-hysteresis two-dimensional relationships were observed between (DiN-AnN in mmHg) and systolic BP (a), dP/dt_max_ (d), dP/dt_max_-RL (e), dP/dt_d_-RL (g), dP/dt_d_–dP/dt_min_ in s (i), diastolic BP (j), pulse BP (k), dP/dt_min_ (m), dP/dt_min_-RL (n), dP/dt_d_–dP/dt_max_ in mmHg (q), anacrotic notch (bb), anacrotic notch delay (dd), anacrotic notch rel. delay (ee), DiN-AnN/dP/dt_min_ (gg), DiN-AnN/dP/dt_max_ (hh), AnN-1Max (ii), dicrotic N. (kk), (DiN-AnN)/dP/dt_min_ (pp) and (DiN-AnN)/dP/dt_max_ (qq) (Figure 4). The remaining relationships between (DiN-AnN in mmHg) and HPs showed hysteresis loops (Figure 4).

Next, we evaluated the non-hysteresis/hysteresis patterns of the two-dimensional cross-relationships during the increase (green line) and decrease (black line) of systolic BP. The non-hysteresis two-dimensional relationships were mostly observed between (DiN-AnN; in mmHg) and HPs (Figure 3). The hysteresis two-dimensional relationships were rarely observed, e.g., between (DiN-AnN; in mmHg) and systolic area (c), dP/dt_max_ (d), or DiN-AnN (ff). Generally, the hysteresis was mostly not observed in the “green–black” region of systolic BP, and even the hysteresis was pronounced in the ‘red–blue’ region of the same cross-relationships, for example, heart rate (b), dP/dtd–dP/dt_max_ (h), or dP/dt_d_ delay (p).

### 2.2. The Cross-Relationships of 34 HPs to the Time Interval between Dicrotic and Anacrotic Notches

**Figure 5:**
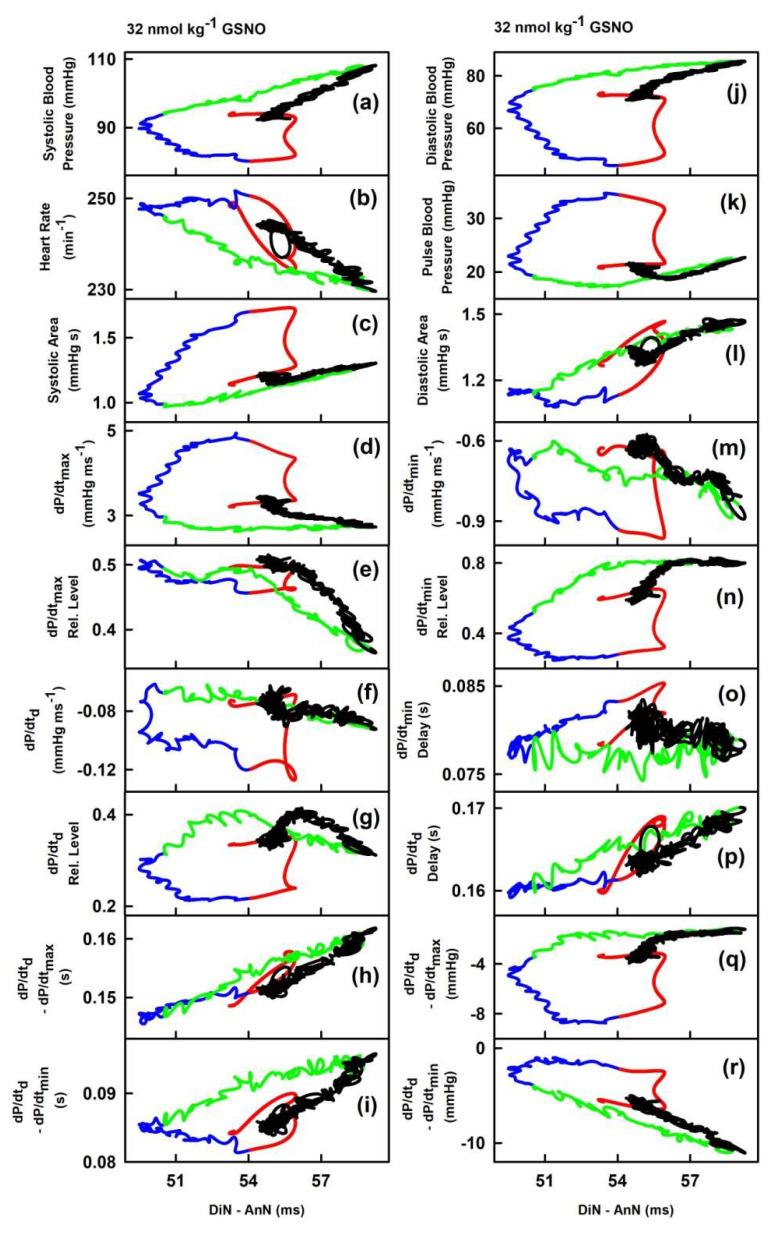

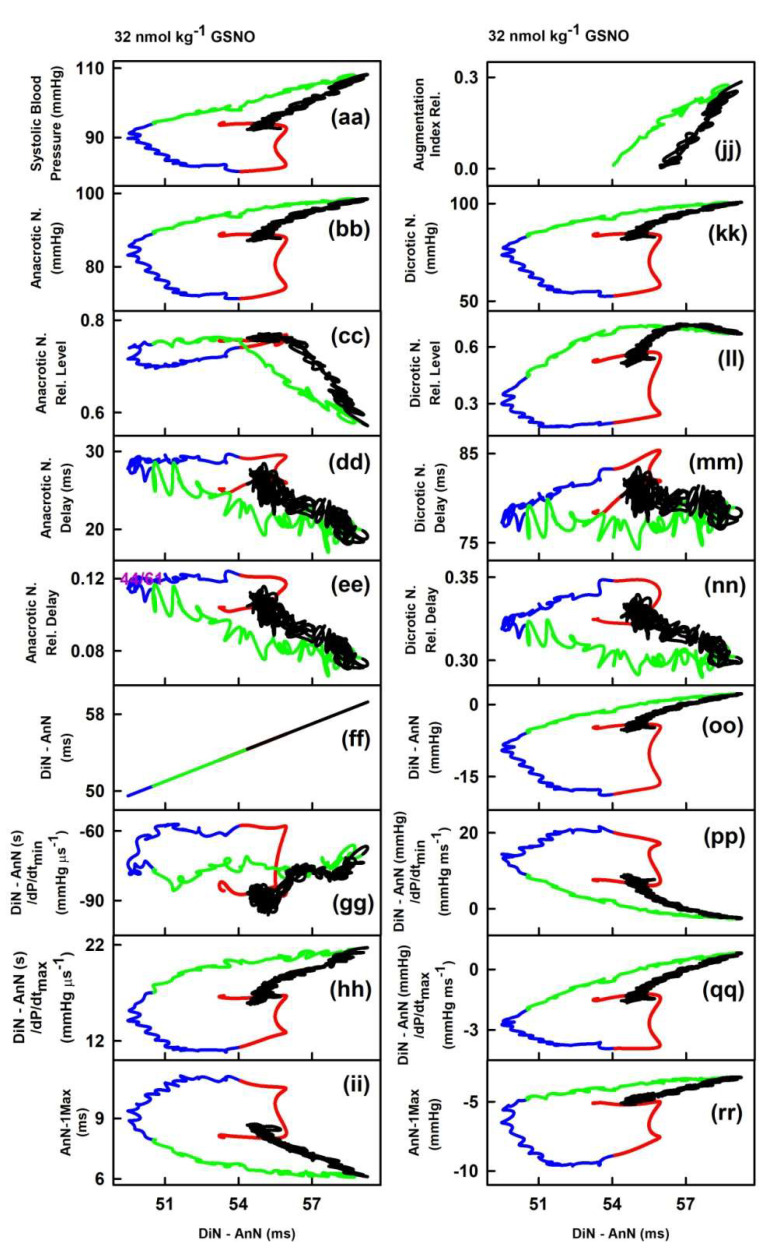
Relationships of HPs to the time interval between dicrotic (DiN) and anacrotic (AnN) notches after the administration of 32 nmol·kg^–1^ GSNO. The colors and time-dependent data correspond to Figure 2. Time period corresponding to the decrease of systolic BP (red), increase of systolic BP to the control value (blue), further increase of systolic BP to maximum (green), and decrease of systolic BP to the control value (black). The hysteresis was arbitrary defined as HPs-(DiN-AnN in ms) loop > 3 ms of DiN-AnN.

In order to observe the two-dimensional cross-relationships of the 34 HPs to the time interval of DiN and AnN (DiN-AnN in ms), the experimental data of the time-dependent changes of DiN-AnN in ms (Figure 2, plot (ff)) were used as x-axis values for Figure 5 and for other six rats, see Figure S3. As in Figure 3, we omitted time dimension and present two dimensional cross-relationships only. The time in the plots of Figure 5 starts at the time interval point 53.3 ms and continues to the point 54 ms (red line), then to 50.4 ms (blue line), further to 58.8 ms (green line), and finally to the point 54.6 ms (black line).

The cross-relationships revealed that changes of DiN-AnN (in ms) during the decrease and increase of systolic BP (Figure 5, the first and the second phases, red and blue lines) are not connected to any of the other 34 HPs. Similarly, changes of DiN-AnN (in ms) during the further increase and final decrease (the third and fourth phases, green and black lines) of systolic BP are not connected to any of the other 34 HPs. The two-dimensional cross-relationships of HPs to the time interval between dicrotic and anacrotic notches (DiN-AnN in ms) showed hysteresis for all HPs. It is noticed that the changes of DiN-AnN (ms) during decreased BP (red lines, Figure 2) for all HPs were minor, they were within time intervals ~3 ms.

### 2.3. Cross-Relathionships of DiN-AnN to 34 Rat HPs for Conditions of Increase/Decreased NO Bioavailability

**Figure 6:**
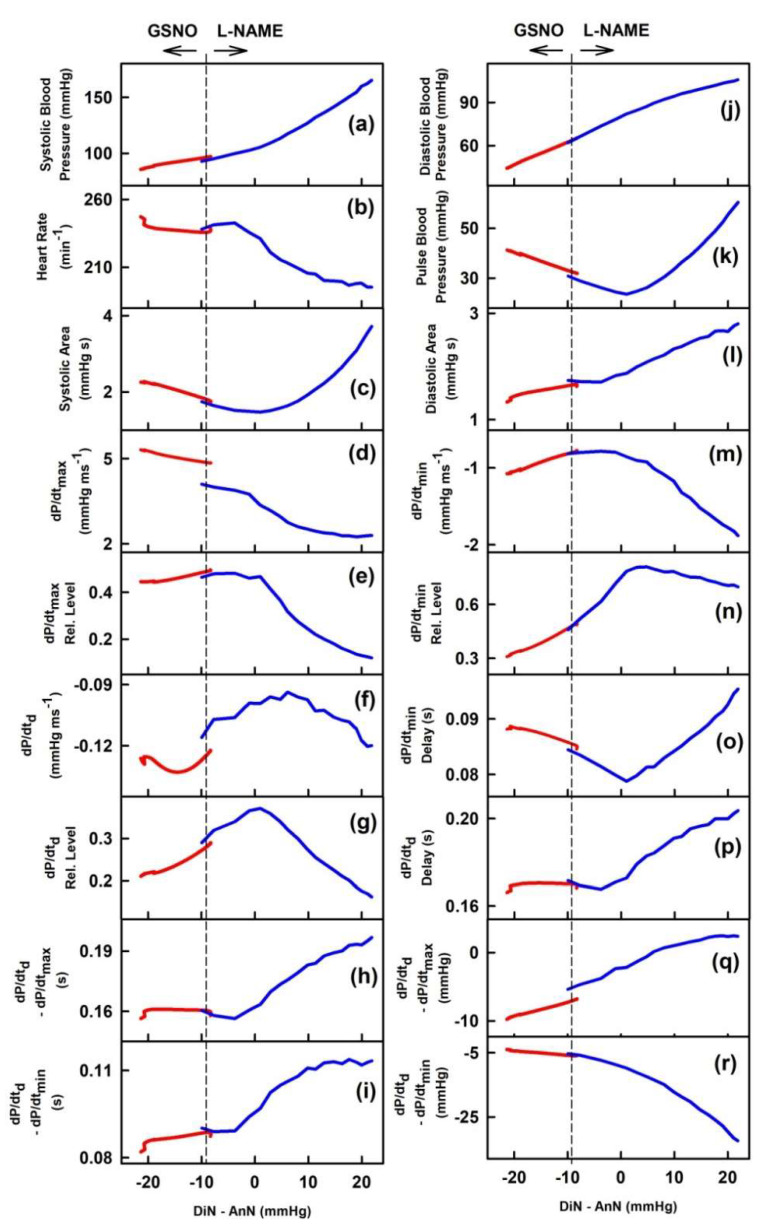

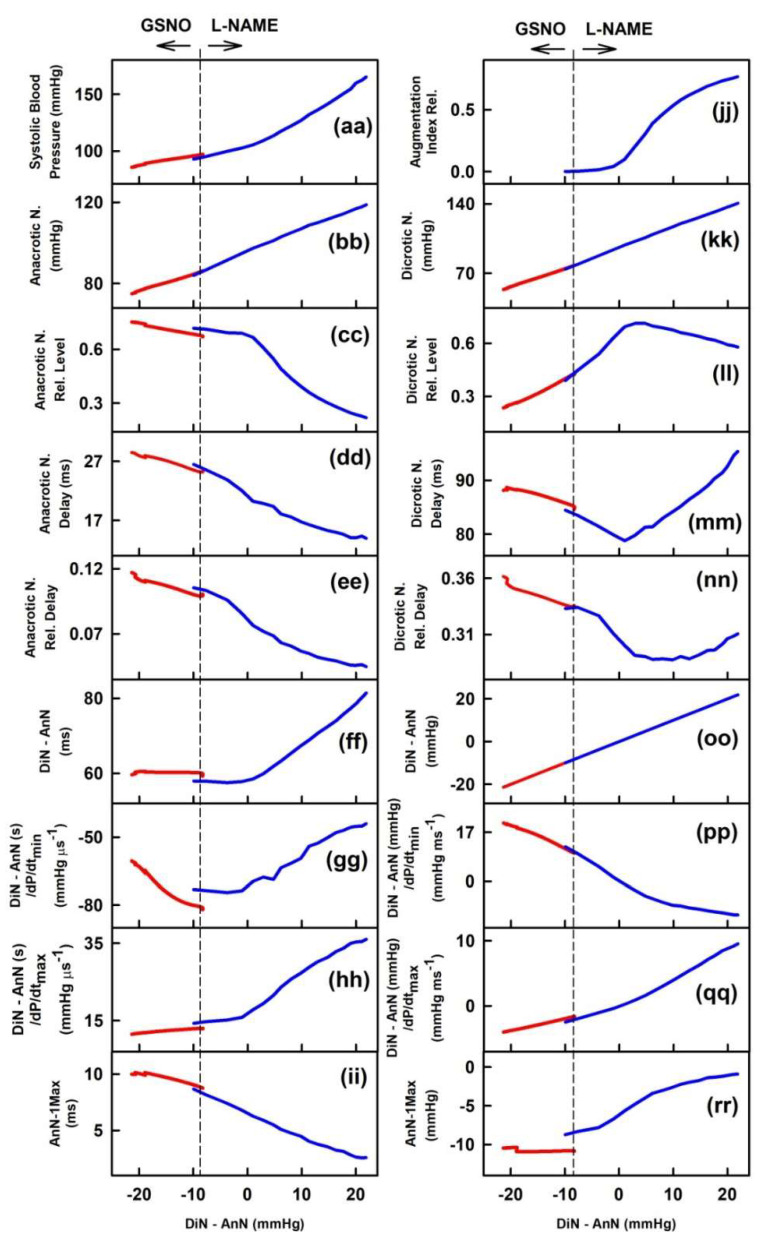
Two-dimensional cross-relationships of DiN-AnN pressure difference (in mmHg) to 34 rat HPs for conditions of increased/decreased NO bioavailability. The increase of NO bioavailability was caused by i.v. administration of 32 nmol·kg^−1^ GSNO (red line; average from 10 experiments; data calculated from Figure 6 published in [22]). Red line was calculated from the time segment during which BP decreased after GSNO administration. The decrease of NO bioavailability after i.v. administration of 25 mg·kg^−1^ l-NAME (blue line; average from six experiments; data calculated from Figure 3 published in [23]) is shown. Blue line was calculated from the time segment during which BP increased after l-NAME administration. Arrows indicate the time direction after GSNO and l-NAME administration.

It was of interest to know how the data of the DiN-AnN cross-relationships during the increase of NO bioavailability are related to the data of the DiN-AnN cross-relationships during the decrease of NO bioavailability. To address this question, the two-dimensional cross-relationships of DiN-AnN to 34 rat HPs for conditions of decreased NO bioavailability by l-NAME were evaluated from the published APW data [23]. The evaluated data were pooled together with the data obtained from APW in the condition of increased NO bioavailability induced by GSNO [22]. Figure 6 shows the two-dimensional cross-relationships of DiN-AnN pressure difference (mmHg) to 34 rat HPs for conditions of increased/decreased NO bioavailability caused by i.v. administration of GSNO and l-NAME, respectively. Each cross-relationship showed different pattern. The DiN-AnN pressure difference (mmHg) during decrease of NO bioavailability mostly followed on the data obtained during increased NO bioavailability for all cross-relationships. Notably, several cross-relationships were bell shaped with maximum or minimum mostly in the region of decreased NO bioavailability (Figure 6).

**Figure 7:**
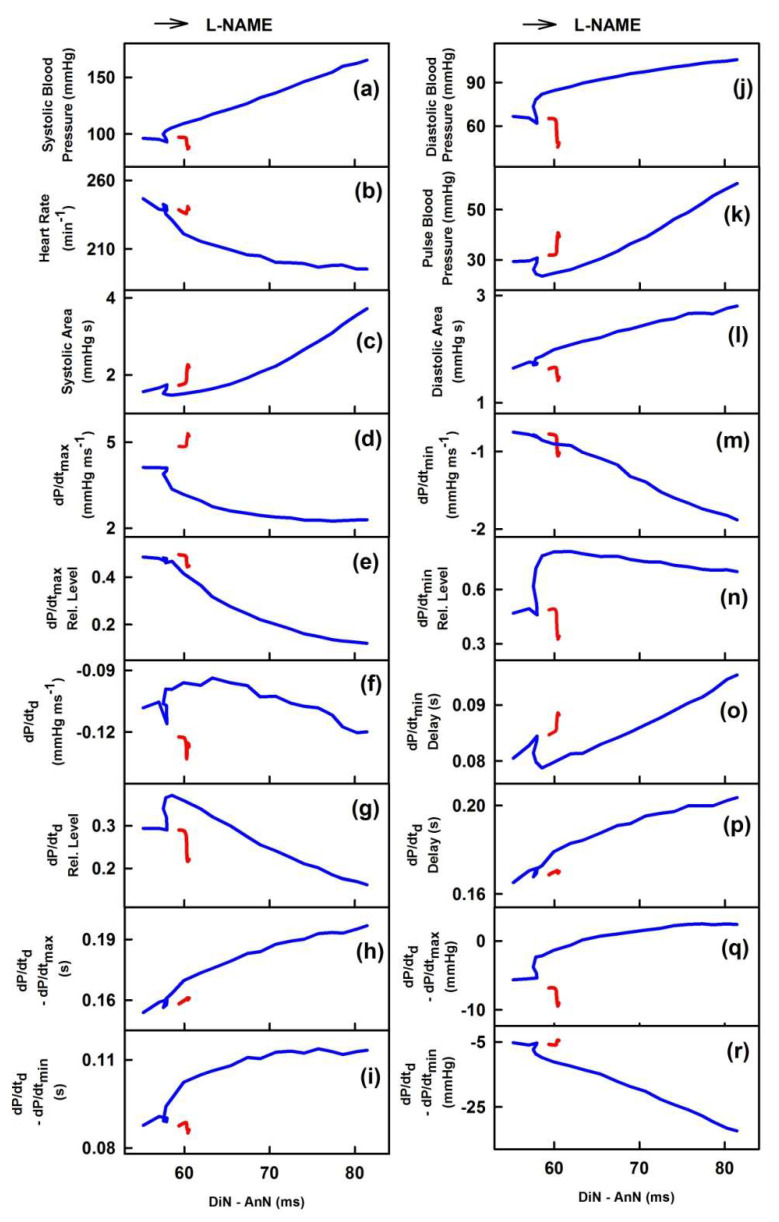

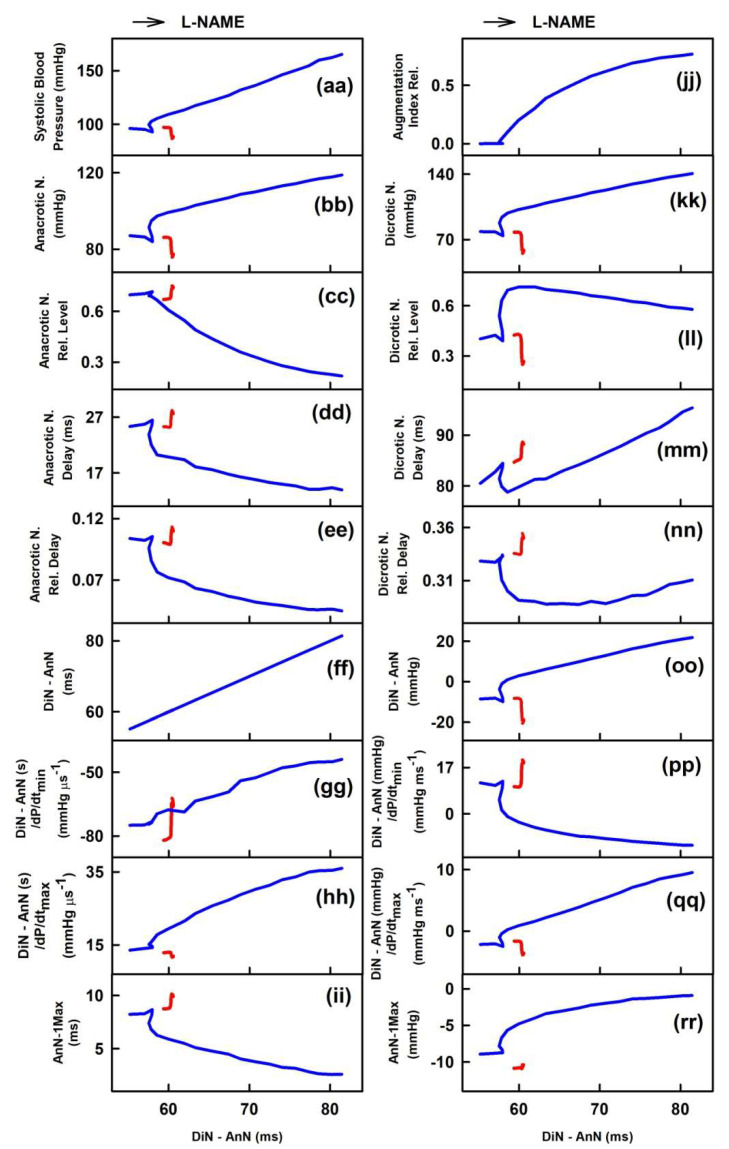
Two-dimensional cross-relationships of DiN-AnN time interval difference (in ms) to 34 rat HPs for conditions of increased/decreased NO bioavailability. The increase of NO bioavailability was caused by i.v. administration of 32 nmol·kg^−1^ GSNO (red line; average from 10 experiments; data calculated from Figure 6 published in [22]). Red line was calculated from the time segment during which BP decreased after GSNO administration. The decrease of NO bioavailability was caused by i.v. administration of 25 mg·kg^−1^ l-NAME (blue line; average from 6 experiments; data calculated from Figure 3 published in [23]). Blue line was calculated from the time segment during which BP increased after l-NAME administration. Arrow indicates the time direction after l-NAME administration.

Figure 7 shows the two-dimensional cross-relationships of the time interval DiN-AnN (ms) to 34 rat HPs for conditions of increased/decreased NO bioavailability caused by i.v. administration of GSNO and l-NAME, respectively. The time interval DiN-AnN (ms) during decrease of NO bioavailability did not follow on the data during increase of NO bioavailability for all cross-relationships. It is noticed that changes of the time interval DiN-AnN (ms) during the decrease BP induced by GSNO (e.g., red line in Figure 5) were within ~3 ms, whereas the changes DiN-AnN (ms) during the increased BP induced by l-NAME [23] were within ~50 ms.

## 3. Discussion

This work is a continuation of our previous studies examining the hypothesis that it is possible to characterize cardiovascular system in many patho-physiological conditions just from the shape of APW [22,23,27]. For the characterization of the cardiovascular system, numerous “patterns” of HPs and their cross-relationships derived from APW must be known in order to create a database of unique patterns for various cardiovascular conditions. In our previous work, we defined 35 HPs and elaborated protocol to obtain detailed changes of APW from which 35 HPs were calculated, and described the cross-relationship patterns between systolic BP and 34 HPs, and between augmentation index and 34 HPs in the case of decreased NO bioavailability [23]. The direct/indirect signaling pathways were suggested from the cross-relationship patterns of systolic BP to 34 HPs obtained from anesthetized rats [22]. In the present work, the same records of APW during increase/decrease of NO bioavailability were evaluated to find cross-relationship patterns of 34 HPs to DiN-AnN intervals.

Our study aimed to obtain primary information about patterns of DiN-AnN relations at increased/decreased NO bioavailability. Using the presented approach, one can study numerous cardiovascular active compounds, evaluate their time-dependent effects on 35 HPs, and calculate other derived 595 cross-relationships parameters, in order to obtain compounds “patterns”. This approach may be used in animal models to create a data bank of patterns of cross-relationships of HPs for different cardiovascular conditions. However, the limitation of the present study is that rats are under anesthesia, which may modulate responses of the compounds. Therefore, effects of different types of anesthesia on HPs should be studied as well.

In the given model conditions of anesthetized rats and increase of NO bioavailability by GSNO, the patterns of HPs to DiN-AnN (in mmHg) were significantly diverse in comparison to the patterns of HPs to DiN-AnN (in ms) (Figure 3; Figure 5). This indicates that different signaling pathways, or their parts, are responsible for the modulation of the DiN-AnN pressure interval and for the modulation of the DiN-AnN time interval. More studies are needed to determine the nature of these signaling pathways. The non-hysteresis two-dimensional relationships between the DiN-AnN (in mmHg) and other 19 HPs (Figure 4) suggest that these HPs, i.e., their signaling pathways, responding to NO concentration, are probably directly connected. The hysteresis relationships between the DiN-AnN (in mmHg) and the remaining 14 HPs (Figure 4) suggest that the signaling pathways of these HPs are indirectly connected. On the other hand, only the hysteresis relationships were observed between the DiN-AnN (in ms) and other 34 HPs (Figure 5), indicating that in the condition of transient increase of NO bioavailability, signaling pathway(s) regulating DiN-AnN (in ms) are not directly connected to any other HP studied.

The increase phase of systolic BP after GSNO administration (Figure 2, green and blue lines) could be associated with a sympathetic reflex response, as observed for several vasoactive substances such as endothelin, urotensin, and apelin [28,29,30,31]. Interestingly enough, comparing the same cross-relationships, the hysteresis was mostly not observed in the “green–black” region of BP, even though it was pronounced in the “red–blue” region. These results may indicate that in contrast to increased NO bioavailability, the signaling pathways regulating the DiN-AnN intervals and other HPs during symphatetic reflex are directly connected.

Interestingly, we found a connection between the correlations of HPs to DiN-AnN in mmHg during increase of NO induced by GSNO and the correlations of HPs to DiN-AnN in mmHg during decrease of NO induced by l-NAME (Figure 6), suggesting that DiN-AnN in mmHg may be regulated by the same pathway(s). In contrast, no connections were found between the correlations of HPs to time interval DiN-AnN in ms during increased/decreased NO bioavailability (Figure 7). This may indicate that different pathway(s) regulate DiN-AnN in ms during decrease and increase of NO bioavailability.

Accumulating evidence suggests that BP and time position of DiN reflect the state of the cardiovascular system. Changes of DiN position has been reported in patients with various cardiovascular complications, such as hypertension, diabetes, and aortic stenosis, and they were modulated by NO bioavailability [14,15,16,17,32]. However, we suppose that recording of AnN and evaluating the DiN-AnN intervals in mmHg and in ms, together with characterization of the cross-relationships between DiN-AnN intervals and other HPs, will add better informative value of the cardiovascular condition than the evaluation of DiN alone.

## 4. Materials and Methods

### 4.1. Ethical Approval

All procedures were approved (10 August 2017) by the State Veterinary and Food Administration of the Slovak Republic (C.k. Ro 3123/17-221; SK UCH 01017) according to the guidelines from Directive 2010/63/EU of the European Parliament. The procuration of animals, the husbandry, and the experiments conform to the “European Convention for the Protection of Vertebrate Animals used for Experimental and other Scientific Purposes” (Council of Europe No 123, Strasbourg 1985). Experiments were carried out as previously described [22,23].

### 4.2. Animals, APW Measurement, and Data Evaluation

For the evaluation of the anacrotic/dicrotic cross-relationships, the recorded data of APW published in [22] were used. The data were obtained from male Wistar rats (*n* = 16; 340 ± 40 g) anesthetized with Zoletil 100 (tiletamine + zolazepam, 80 mg·kg^−1^, i.p.) and Xylazine (5 mg·kg^−1^, i.p.). The right jugular vein of anesthetized Wistar rats was cannulated for i.v. administration of compounds. After the stabilization of systolic BP (10–15 min), 32 nmol·kg^−1^ GSNO or 25 mg·kg^−1^ l-NAME prepared in 0.9% saline solution was administered into the right jugular vein (500 µL·kg^−1^) over 15 s period. The compounds administration started 40 ± 10 min after the onset of anesthesia. The left common carotid artery (*arteria carotis communis*) was cannulated to insert the fiber optic micro-catheter pressure transducers (FISO LS 2F Harvard Apparatus, Holliston, MA, USA) connected to the FISO Series Signal Conditioners to measure APW. The analogue signal was filtered by lowpass filter 2.5 kHz, digitalized at 10 kHz, and analyzed by the application wrote in MATLAB (the MathWorks, Inc., Natick, MA, USA) to identify and analyze ten points (a–j) of APW (Supplementary Materials Figure S1). For the purpose of this work, only two points are described (Figure 1). Detailed definition and abbreviation of the 35 HP calculated from APW are described in [22,23].

## 5. Conclusions

Our work explores changes in APW in conditions of transient increase/decrease of NO bioavailability and presents numerous original data characterizing APW by BP and time positions of DiN-AnN in anesthetized rat. The non-hysteresis/hysteresis two-dimensional cross-relationships “patterns” of DiN-AnN to other 34 HPs revealed their direct or indirect signaling pathways connections. The patterns of HPs to DiN-AnN (in mmHg) were significantly diverse in comparison to the patterns of HPs to DiN-AnN (in ms), indicating that different signaling pathways are responsible for the modulation of BP and time intervals. The DiN-AnN cross-relationship “patterns” can serve for additional definition of the conditions of transient increase/decrease of NO in the cardiovascular system. However, to determine which of the DiN-AnN cross-relationship patterns are “unique” for NO signaling, more studies are needed. The results revealed some of the detailed GSNO and l-NAME actions in the cardiovascular system that may contribute to the understanding of biological effects of natural substances modulating NO production and/or NO signaling pathways.

From a clinical perspective, our approach and “pattern” data from the animal research can be used as basic research information to start similar studies on humans. Even though current non-invasive techniques have lower time/pressure resolution in comparison to invasive fiber optic micro-catheter pressure transducer, it would be of interest to look for cross-relationships between HPs obtained from human APW at different patho-physiological conditions and to look for “unique patterns” of particular cardiovascular conditions.

## Supplementary Materials

Supplementary materials can be found at https://www.mdpi.com/1422-0067/21/18/6685/s1.

## Abbreviations

AnN	Anacrotic notch
APW	Arterial pulse waveform
BP	Blood pressure
DiN	Dicrotic notch
GSNO	S-nitrosoglutathione
HPs	Hemodynamic parameters
l-NAME	N(ω)-nitro-l-arginine methyl ester
NO	Nitric oxide

## References

[1] Forte M., Conti V., Damato A., Ambrosio M., Puca A.A., Sciarretta S., Frati G., Vecchione C., Carrizzo A. (2016). Targeting Nitric Oxide with Natural Derived Compounds as a Therapeutic Strategy in Vascular Diseases. Oxid. Med. Cell. Longev..

[2] Schmitt C.A., Dirsch V.M. (2009). Modulation of endothelial nitric oxide by plant-derived products. Nitric Oxide Biol. Chem..

[3] Barnett S.D., Buxton I.L.O. (2017). The role of S-nitrosoglutathione reductase (GSNOR) in human disease and therapy. Crit. Rev. Biochem. Mol. Biol..

[4] Duarte J., Francisco V., Perez-Vizcaino F. (2014). Modulation of nitric oxide by flavonoids. Food Funct..

[5] Jiang H., Torregrossa A.C., Parthasarathy D.K., Bryan N.S. (2012). Natural product nitric oxide chemistry: New activity of old medicines. Evid. Based Complementary Altern. Med. eCAM.

[6] Smith B.C., Marletta M.A. (2012). Mechanisms of S-nitrosothiol formation and selectivity in nitric oxide signaling. Curr. Opin. Chem. Biol..

[7] Broniowska K.A., Diers A.R., Hogg N. (2013). S-nitrosoglutathione. Biochim. Biophys. Acta.

[8] Kopincová J., Púzserová A., Bernátová I. (2012). l-NAME in the cardiovascular system-nitric oxide synthase activator?. Pharmacol. Rep..

[9] Vlachopoulos C., O’Rourke M. (2000). Genesis of the normal and abnormal arterial pulse. Curr. Probl. Cardiol..

[10] Stoner L., Young J.M., Fryer S. (2012). Assessments of arterial stiffness and endothelial function using pulse wave analysis. Int. J. Vasc. Med..

[11] Lekakis J.P., Zakopoulos N.A., Protogerou A.D., Papaioannou T.G., Kotsis V.T., Pitiriga V., Tsitsirikos M.D., Stamatelopoulos K.S., Papamichael C.M., Mavrikakis M.E. (2005). Arterial stiffness assessed by pulse wave analysis in essential hypertension: Relation to 24-h blood pressure profile. Int. J. Cardiol..

[12] Avolio A.P., Butlin M., Walsh A. (2010). Arterial blood pressure measurement and pulse wave analysis--their role in enhancing cardiovascular assessment. Physiol. Meas..

[13] Žikić D. (2017). A mathematical model of pressure and flow waveforms in the aortic root. Eur. Biophys. J..

[14] Nier B.A., Harrington L.S., Carrier M.J., Weinberg P.D. (2008). Evidence for a specific influence of the nitrergic pathway on the peripheral pulse waveform in rabbits. Exp. Physiol..

[15] Rafati M., Havaee E., Moladoust H., Sehhati M. (2017). Appraisal of different ultrasonography indices in patients with carotid artery atherosclerosis. EXCLI J..

[16] Hao Y., Cheng F., Pham M., Rein H., Patel D., Fang Y., Feng Y., Yan J., Song X., Yan H. (2019). A Noninvasive, Economical, and Instant-Result Method to Diagnose and Monitor Type 2 Diabetes Using Pulse Wave: Case-Control Study. JMIR mHealth uHealth.

[17] Marais L., Pernot M., Khettab H., Tanter M., Messas E., Zidi M., Laurent S., Boutouyrie P. (2019). Arterial Stiffness Assessment by Shear Wave Elastography and Ultrafast Pulse Wave Imaging: Comparison with Reference Techniques in Normotensives and Hypertensives. Ultrasound Med. Biol..

[18] Klein L.W., Shahrrava A. (2019). The Incisura. Cardiol. Rev..

[19] Munir S., Guilcher A., Kamalesh T., Clapp B., Redwood S., Marber M., Chowienczyk P. (2008). Peripheral augmentation index defines the relationship between central and peripheral pulse pressure. Hypertension.

[20] Klocke R., Cockcroft J.R., Taylor G.J., Hall I.R., Blake D.R. (2003). Arterial stiffness and central blood pressure, as determined by pulse wave analysis, in rheumatoid arthritis. Ann. Rheum. Dis..

[21] Takazawa K., Kobayashi H., Shindo N., Tanaka N., Yamashina A. (2007). Relationship between radial and central arterial pulse wave and evaluation of central aortic pressure using the radial arterial pulse wave. Hypertens Res..

[22] Misak A., Kurakova L., Berenyiova A., Tomasova L., Grman M., Cacanyiova S., Ondrias K. (2020). Patterns and Direct/Indirect Signaling Pathways in Cardiovascular System in the Condition of Transient Increase of NO. BioMed Res. Int..

[23] Kurakova L., Misak A., Tomasova L., Cacanyiova S., Berenyiova A., Ondriasova E., Balis P., Grman M., Ondrias K. (2020). Mathematical relationships of patterns of 35 rat haemodynamic parameters for conditions of hypertension resulting from decreased nitric oxide bioavailability. Exp. Physiol..

[24] Politi M.T., Ghigo A., Fernández J.M., Khelifa I., Gaudric J., Fullana J.M., Lagrée P.Y. (2016). The dicrotic notch analyzed by a numerical model. Comput. Biol. Med..

[25] Wang A., Yang L., Wen W., Zhang S., Hao D., Khalid S.G., Zheng D. (2018). Quantification of radial arterial pulse characteristics change during exercise and recovery. J. Physiol. Sci..

[26] Wang A., Yang L., Liu C., Cui J., Li Y., Yang X., Zhang S., Zheng D. (2015). Athletic differences in the characteristics of the photoplethysmographic pulse shape: Effect of maximal oxygen uptake and maximal muscular voluntary contraction. BioMed Res. Int..

[27] Kristek F., Grman M., Ondrias K. (2019). In Vivo Measurement of H(2)S, Polysulfides, and “SSNO(-) Mix”-Mediated Vasoactive Responses and Evaluation of Ten Hemodynamic Parameters from Rat Arterial Pulse Waveform. Methods Mol. Biol..

[28] King A.J., Pfeffer J.M., Pfeffer M.A., Brenner B.M. (1990). Systemic hemodynamic effects of endothelin in rats. Am. J. Physiol..

[29] Gardiner S.M., March J.E., Kemp P.A., Bennett T. (2004). Bolus injection of human UII in conscious rats evokes a biphasic haemodynamic response. Br. J. Pharmacol..

[30] Charles C.J., Rademaker M.T., Richards A.M. (2006). Apelin-13 induces a biphasic haemodynamic response and hormonal activation in normal conscious sheep. J. Endocrinol..

[31] Yang S.C., Guo Y.J., Yu F.Y., Chen L.L., Li W.Y., Ji E.S. (2018). Bosentan ameliorates hypertension in rats exposed to chronic intermittent hypoxia through inhibiting renal sympathetic nerve activity. Sheng Li Xue Bao.

[32] Schmidt R., Weidner C., Schmelz M. (2011). Time course of acetylcholine-induced activation of sympathetic efferents matches axon reflex sweating in humans. J. Peripher Nerv. Syst..

