# Supplementary material for: Characterization of Rat Cardiovascular System by Anacrotic/Dicrotic Notches in the Condition of Increase/Decrease of NO Bioavailability
Source: Int J Mol Sci. 2020 Sep 12;21(18):6685. doi: 10.3390/ijms21186685 (PMC7555952; doi:10.3390/ijms21186685)
Supplement: Supplementary file 1 [file ijms-21-06685-s001.pdf]

**Supplementary Materials**

**To**

# Characterization of rat cardiovascular system by anacrotic/dicrotic notches in the condition of increase/decrease of NO bioavailability

**Lenka Tomasova [1], Anton Misak [1], Lucia Kurakova [1,2], Marian Grman [1] and Karol Ondrias [1,*]**

[1] Institute of Clinical and Translational Research, Biomedical Research Center, Slovak Academy of Sciences, Dubravska cesta 9, 845 05 Bratislava, Slovak Republic; lenka.tomasova@savba.sk (L.T.); anton.misak@savba.sk (A.M.); marian.grman@savba.sk (M.G.); karol.ondrias@savba.sk (K.O.)

[2] Department of Pharmacology and Toxicology, Faculty of Pharmacy, Comenius University, Bratislava, Slovak Republic; kurakova4@uniba.sk (L.K.)

* Correspondence: karol.ondrias@savba.sk; Tel.: +421-908577943

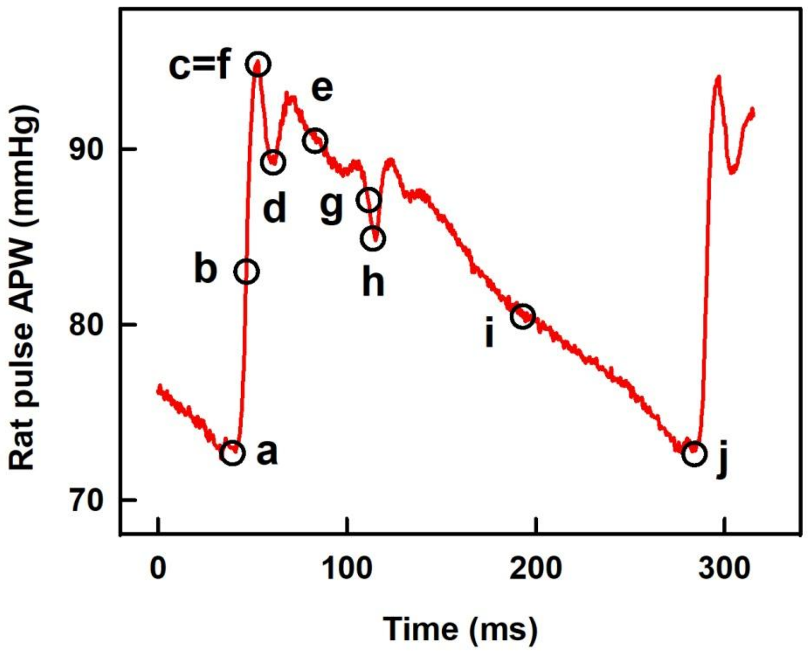


**Figure S1.** Ten points, **a – j**, on rat arterial pulse waveform (APW).

**Description of 35 hemodynamic parameters (HPs) from APW.**

Blood pressure and time position of ten points, **a – j,** on APW were used to define and calculate specific HP (Figure S1). For more details see Kurakova et al. 2020 (DOI: 10.1113/EP088148) and Misak et al. 2020 (doi.org/10.1155/2020/6578213).

Plot (a): Systolic blood pressure in mmHg; point **c** or **f**.

Plot (b): Heart rate in $min^{-1}$; 60 / (**j** – **a**); (**j** – **a**) represents time interval between **a** and **j**, **a** and **j** are two reference points to diastolic BP value.

Plot (c): Systolic area in mmHg s; integral BP of **a** to **h**; **h** refers to BP at the dicrotic notch (dicrotic BP).

Plot (d): $dP/dt_{max}$ in mmHg $ms^{-1}$; maximum derivative at the point **b**; P is BP in mmHg.

Plot (e): $dP/dt_{max}$ relative level; relative level (shortly RL) of point **b**; (**b** – **a**) / (**c** (or **f**) – **a**) in mmHg/mmHg (dimensionless).

Plot (f): $dP/dt_d$ in mmHg $ms^{-1}$; negative maximum derivative at the point **i**; the point **i** is the BP at the middle of the time interval between **h** and **j**.

Plot (g): $dP/dt_d$ relative level, relative level of point **i**; (**i** – **a**) / (**c** (or **f**) – **a**) in mmHg/mmHg (dimensionless).

Plot (h): $dP/dt_d$ – $dP/dt_{max}$ in s; time interval between **b** and **i**, $dP/dt_d$ – $dP/dt_{max}$ = (**i** – **b**).

Plot (i): $dP/dt_d - dP/dt_{min}$ in s; time interval between **g** and **i**, $dP/dt_d - dP/dt_{min}$ = (**i** – **g**); $dP/dt_{min}$ is negative maximum derivative at the point **g**.

Plot (j): Diastolic blood pressure in mmHg; the point **a** or **j.**

Plot (k): Pulse BP in mmHg; (**c** – **a**) or (**f** – **a**).

Plot (l): Diastolic area in mmHg s; integral BP of **h** to **j**.

Plot (m): $dP/dt_{min}$ in mmHg $ms^{-1}$; $dP/dt_{min}$ is maximum negative derivative at the point **g**.

Plot (n): $dP/dt_{min}$ relative level, relative level of point **g**; (**g** – **a**) / (**c** (or **f**) – **a**) in mmHg/mmHg (dimensionless).

Plot (o): $dP/dt_{min}$ delay in s; delay in s of point **g**; (**g** – **a**) time interval between **a** and **g**.

Plot (p): $dP/dt_d$ delay in s; delay in s of point **i**; (**i** – **a**) time interval between **a** and **i**.

Plot (q): $dP/dt_d - dP/dt_{max}$ in mmHg; (**i** – **b**) BP difference between **b** and **i**.

Plot (r): $dP/dt_d - dP/dt_{min}$ in mmHg; (**i** – **g**) BP difference between **g** and **i**.

Plot (aa): Systolic blood pressure in mmHg; point **c** or **f**. Plot (aa) is the same as (a).

Plot (bb): Anacrotic notch in mmHg; BP at the point **d**.

Plot (cc): Anacrotic notch relative level; relative level of point **d**; (**d** – **a**) / (**c** (or **f**) – **a**) in mmHg/mmHg (dimensionless).

Plot (dd): Anacrotic notch delay in ms; delay in ms of point **d**; (**d** – **a**) time interval between **a** and **d**.

Plot (ee): Anacrotic notch relative delay; relative delay (shortly RD) of point **d**; (**d** – **a**) / (**j** – **a**) in ms/ms (dimensionless)

Plot (ff): [Dicrotic notch (DiN) in s] – [Anacrotic notch (AnN) in s] in s; (**h** – **d**) time interval between **d** and **h**.

Plot (gg): [(DiN – AnN) in s] / [$dP/dt_{min}$ in mmHg $\mu s^{-1}$]* in s/mmHg $\mu s^{-1}$; (**h** – **d**) / **g**.

Plot (hh): [(DiN – AnN) in s] / [$dP/dt_{max}$ in mmHg $\mu s^{-1}$] in s/mmHg $\mu s^{-1}$; (**h** – **d**) / **b**.

Plot (ii): [AnN in ms] – [1Max (point **c** or the 1th. maximum) in ms] in ms; (**d** – **c**) time interval between **c** and **d**.

Plot (jj): Augmentation index relative; (**f** – **c**) / (**f** – **a**) in mmHg/mmHg (dimensionless).

Plot (kk): Dicrotic notch in mmHg; BP at the point **h**.

Plot (ll): Dicrotic notch relative level; relative level of point **h**; (**h** – **a**) / (**c** (or **f**) – **a**) in mmHg/mmHg (dimensionless).

Plot (mm): Dicrotic notch delay in ms, delay in ms of point **h**; (**h** – **a**).time interval between **a** and **h**.

Plot (nn): Dicrotic notch relative delay; relative delay of point **h**; (**h** – **a**) / (**j** – **a**); in ms/ms (dimensionless)

Plot (oo): [DiN in mmHg] – [AnN in mmHg] in mmHg; (**h** – **d**) BP difference between **d** and **h**;

Plot (pp): [(DiN – AnN) in mmHg] / [$dP/dt_{min}$ in mmHg $ms^{-1}$] in mmHg/mmHg $ms^{-1}$; (**h** – **d**) / **g**;

Plot (qq): [(DiN – AnN) in mmHg] / [$dP/dt_{max}$ in mmHg $ms^{-1}$] in mmHg/mmHg $ms^{-1}$; (**h** – **d**) / **b**.

Plot (rr): [AnN in mmHg] – [1Max (point **c** or the 1th. maximum) in mmHg] in mmHg; (**d** – **c**) BP difference between **c** and **d**.

*Units in plots (gg), (hh), (pp) and (qq) are informative only.

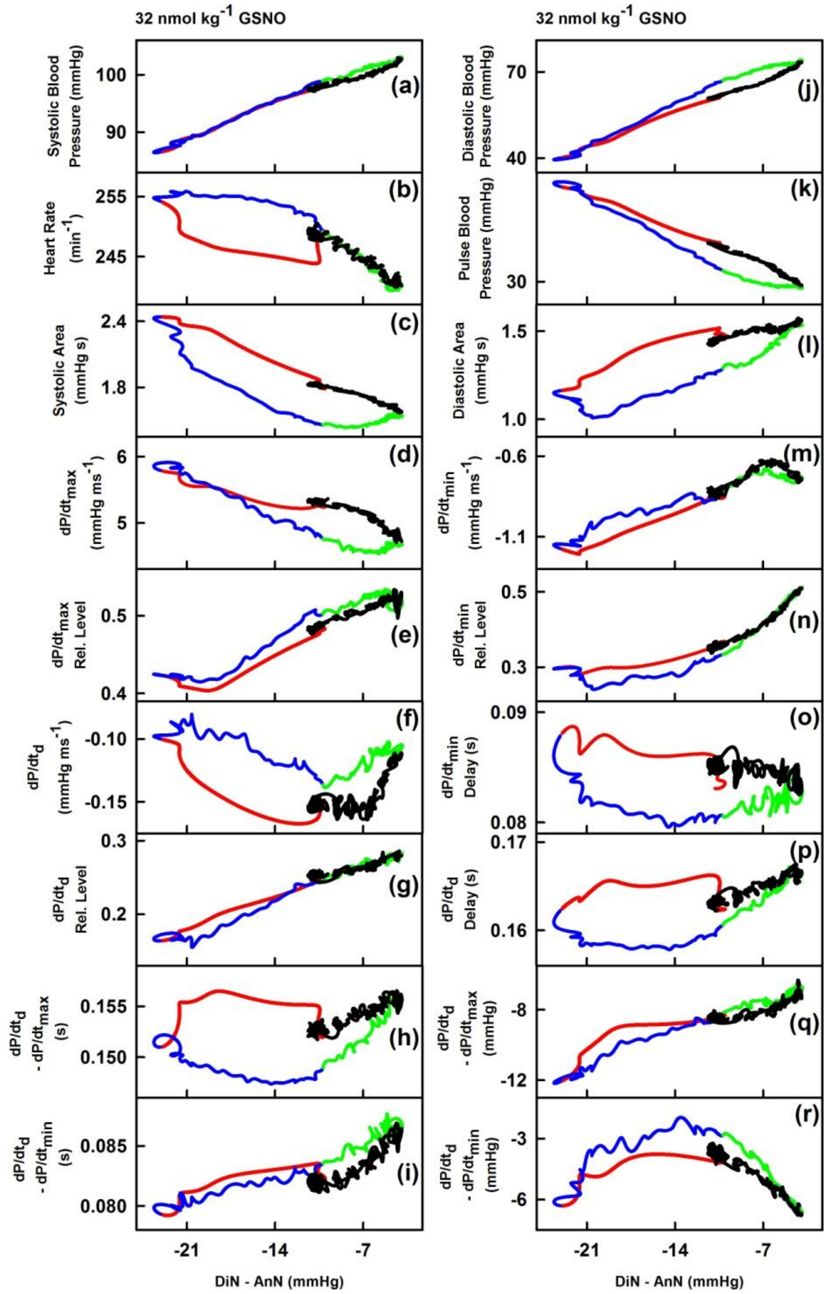

32 nmol kg-1 GSNO
32 nmol kg-1 GSNO
Systolic Blood Pressure (mmHg)
Diastolic Blood Pressure (mmHg)
Heart Rate (min-1)
Pulse Blood Pressure (mmHg)
Systolic Area (mmHg s)
Diastolic Area (mmHg s)
dP/dtmax (mmHg ms-1)
dP/dtmin (mmHg ms-1)
dP/dtmax Rel. Level
dP/dtmin Rel. Level
dP/dtd (mmHg ms-1)
dP/dtmin Delay (s)
dP/dtd Rel. Level
dP/dtd Delay (s)
dP/dtd - dP/dtmax (s)
dP/dtd - dP/dtmax (mmHg)
dP/dtd - dP/dtmin (s)
dP/dtd - dP/dtmin (mmHg)
(a)
(b)
(c)
(d)
(e)
(f)
(g)
(h)
(i)
(j)
(k)
(l)
(m)
(n)
(o)
(p)
(q)
(r)
DiN - AnN (mmHg)
DiN - AnN (mmHg)

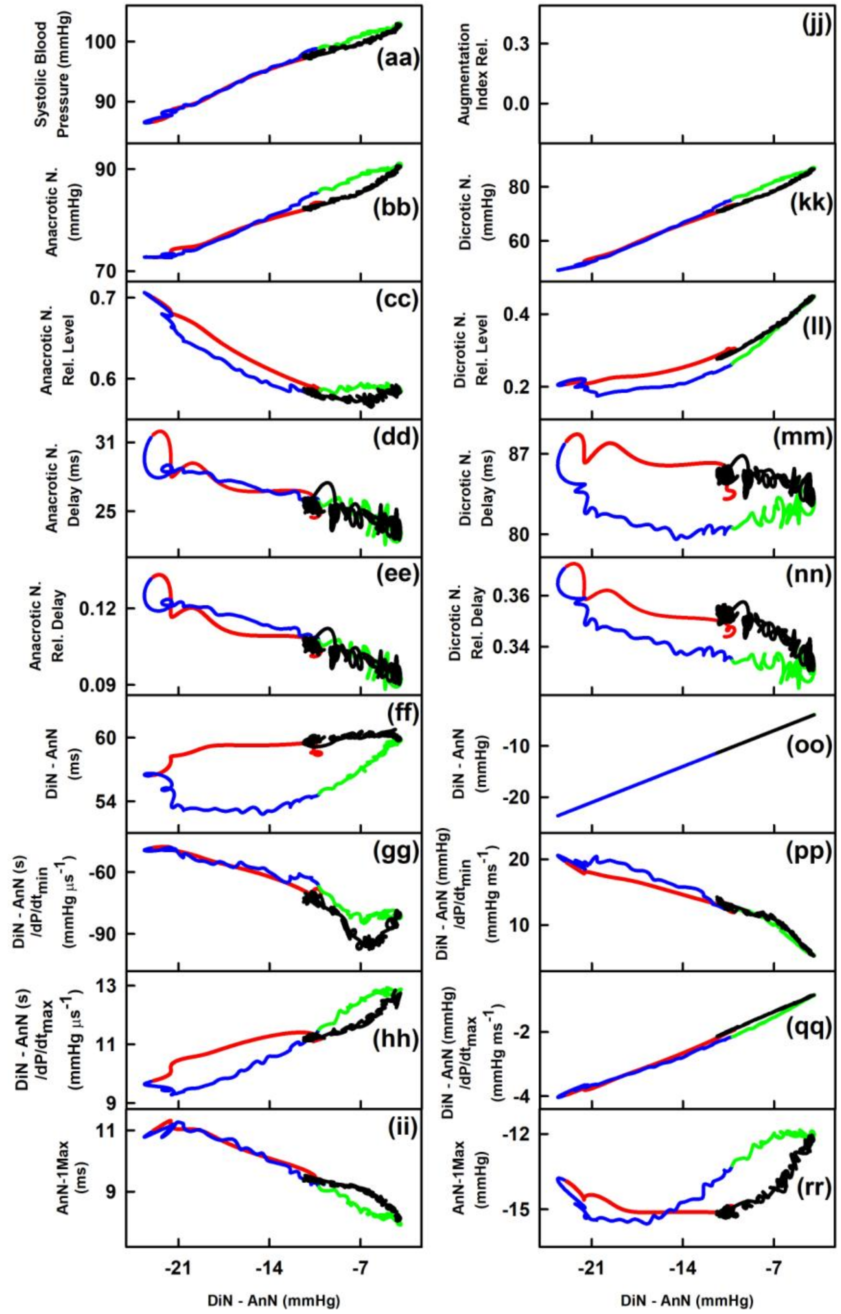


**Figure S2A.** Relationships of HPs to the blood pressure (BP) interval between dicrotic (DiN) and anacrotic (AnN) notches after the administration of 32 nmol kg$^{-1}$ S-nitrosoglutathione (GSNO). The colors and time dependent data correspond to Figure 2. The hysteresis was arbitrary defined as HPs-(DiN-AnN in mmHg) loop > 5 mmHg of DiN-AnN. The non-hysteresis was arbitrary defined as HPs-(DiN-AnN in mmHg) loop ≤ 5 mmHg of DiN-AnN.

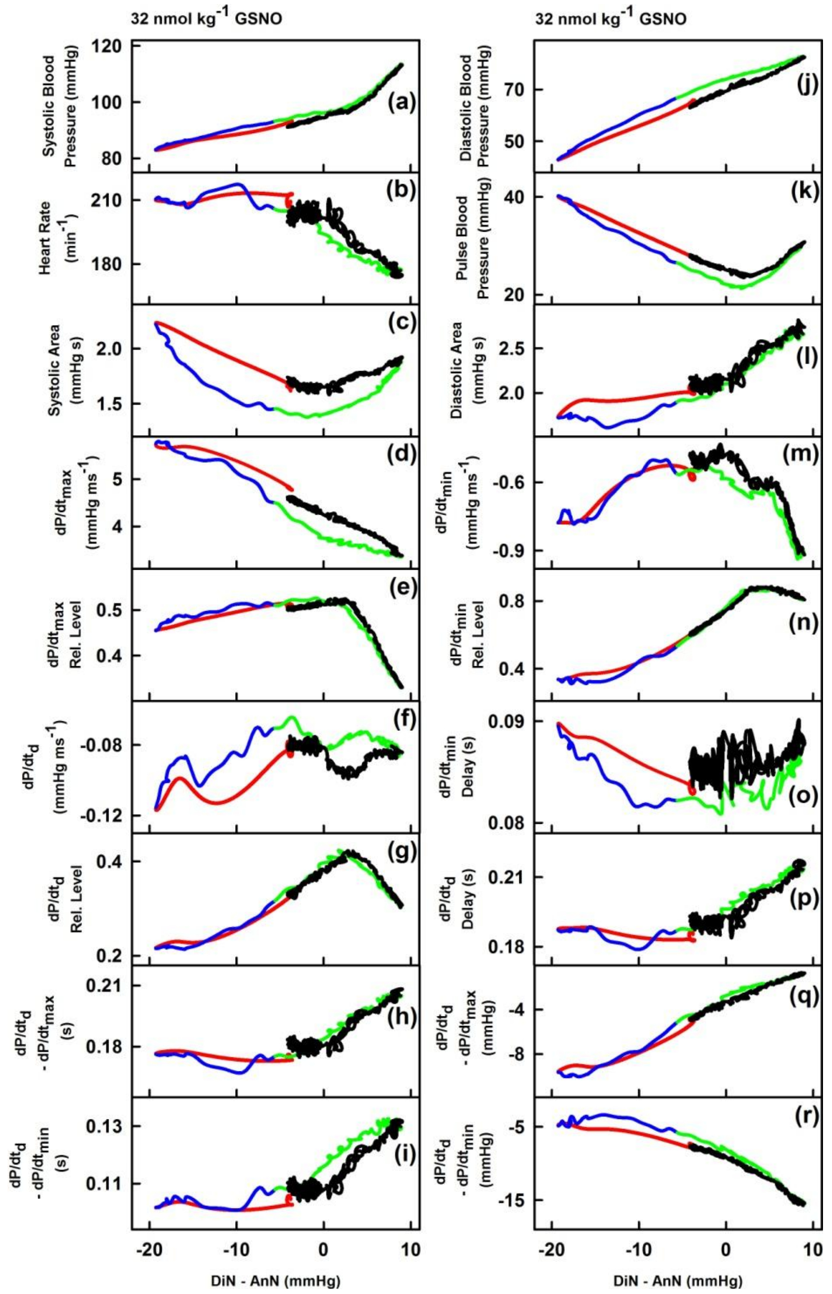
32 nmol kg-1 GSNO
32 nmol kg-1 GSNO
Systolic Blood Pressure (mmHg)
Heart Rate (min-1)
Systolic Area (mmHg s)
dP/dtmax (mmHg ms-1)
dP/dtmax Rel. Level
dP/dtd (mmHg ms-1)
dP/dtd Rel. Level
dP/dtd - dP/dtmax (s)
dP/dtd - dP/dtmin (s)
Diastolic Blood Pressure (mmHg)
Pulse Blood Pressure (mmHg)
Diastolic Area (mmHg s)
dP/dtmin (mmHg ms-1)
dP/dtmin Rel. Level
dP/dtmin Delay (s)
dP/dtd Delay (s)
dP/dtd - dP/dtmax (mmHg)
dP/dtd - dP/dtmin (mmHg)
(a)
(b)
(c)
(d)
(e)
(f)
(g)
(h)
(i)
(j)
(k)
(l)
(m)
(n)
(o)
(p)
(q)
(r)
DiN - AnN (mmHg)
DiN - AnN (mmHg)

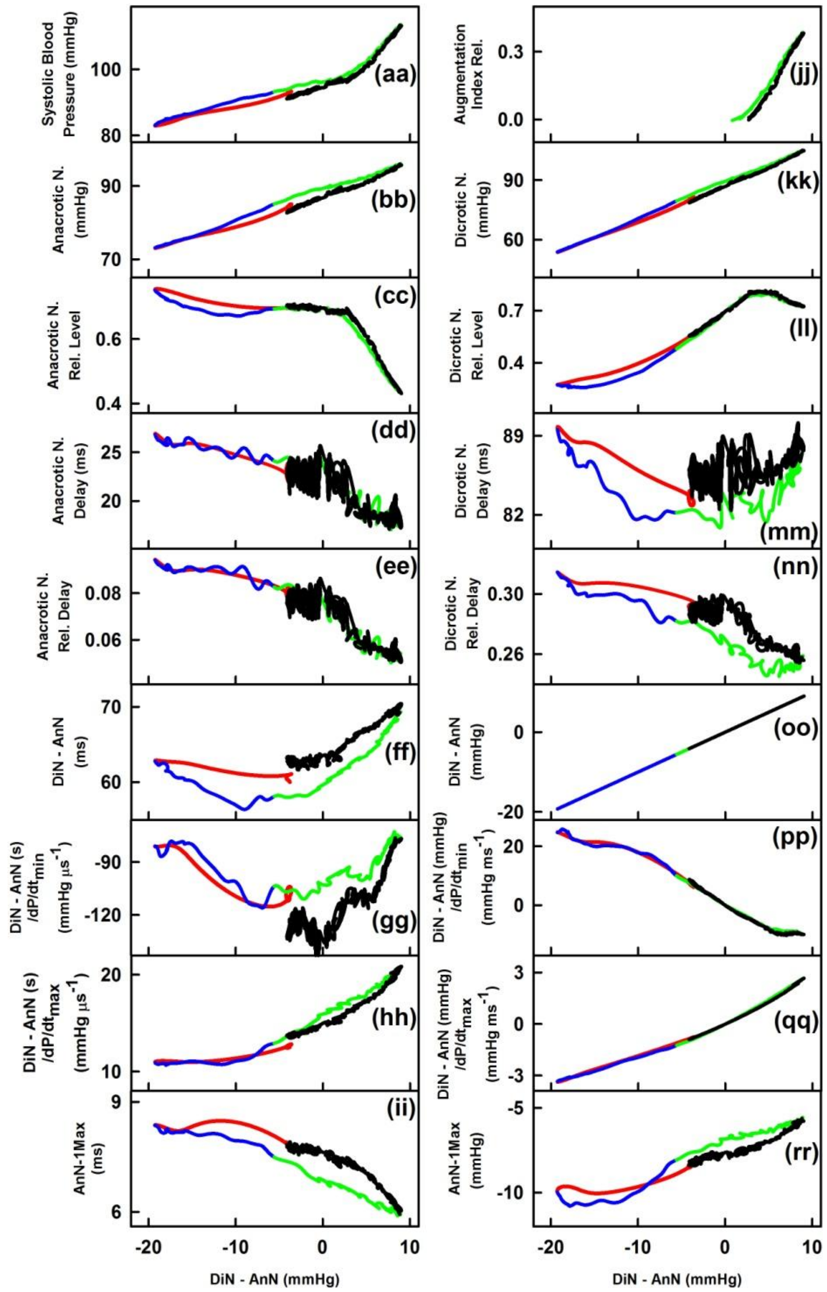


**Figure S2B.** Relationships of HPs to the BP interval between dicrotic (DiN) and anacrotic (AnN) notches after the administration of 32 nmol kg$^{-1}$ GSNO. The colors and time dependent data correspond to Figure 2. The hysteresis was arbitrary defined as HPs-(DiN-AnN in mmHg) loop > 5 mmHg of DiN-AnN. The non-hysteresis was arbitrary defined as HPs-(DiN-AnN in mmHg) loop ≤ 5 mmHg of DiN-AnN.

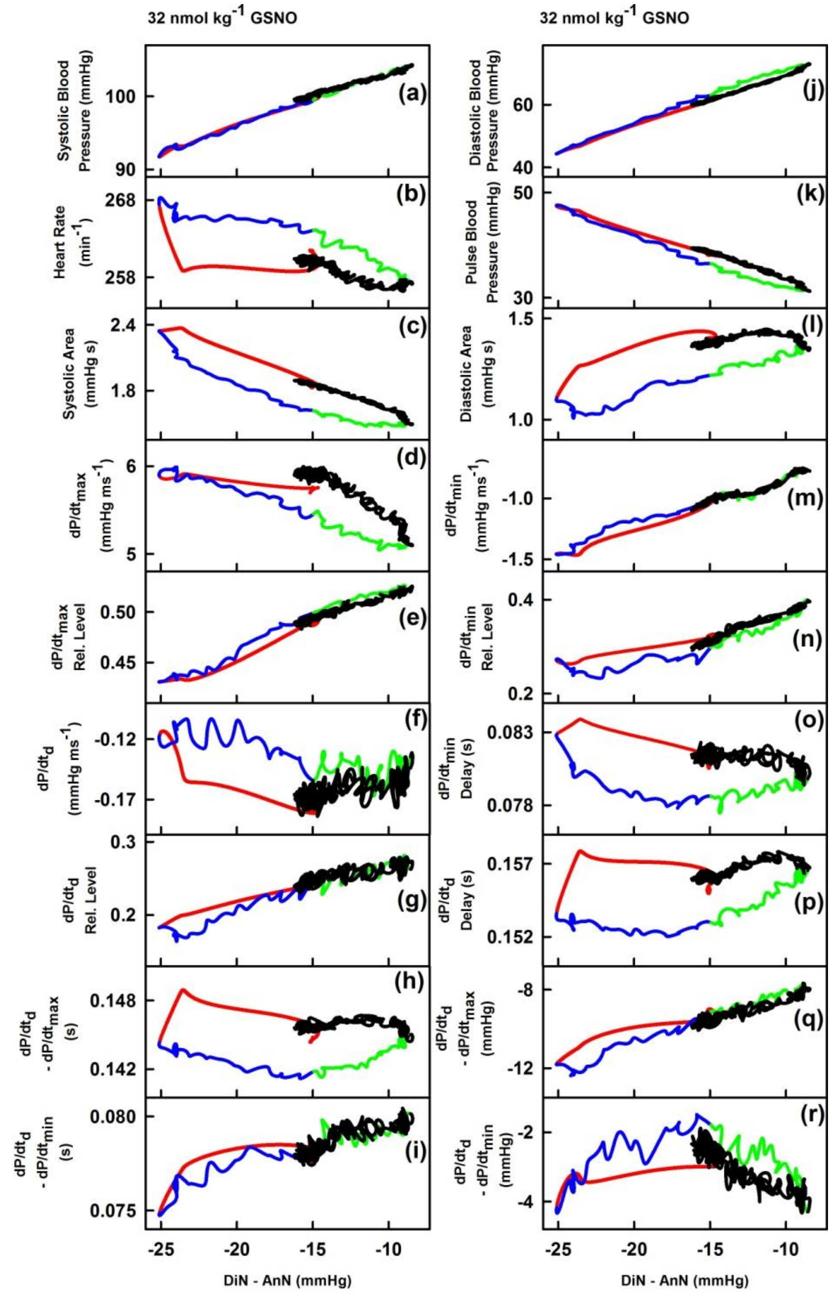
32 nmol kg$^{-1}$ GSNO
32 nmol kg$^{-1}$ GSNO
Systolic Blood Pressure (mmHg)
90
100
(a)
Heart Rate (min$^{-1}$)
258
268
(b)
Systolic Area (mmHg s)
1.8
2.4
(c)
$dP/dt_{max}$ (mmHg ms$^{-1}$)
5
6
(d)
$dP/dt_{max}$ Rel. Level
0.45
0.50
(e)
$dP/dt_d$ (mmHg ms$^{-1}$)
-0.17
-0.12
(f)
$dP/dt_d$ Rel. Level
0.2
0.3
(g)
$dP/dt_d$ - $dP/dt_{max}$ (s)
0.142
0.148
(h)
$dP/dt_d$ - $dP/dt_{min}$ (s)
0.075
0.080
(i)
-25
-20
-15
-10
DiN - AnN (mmHg)
Diastolic Blood Pressure (mmHg)
40
60
(j)
Pulse Blood Pressure (mmHg)
30
50
(k)
Diastolic Area (mmHg s)
1.0
1.5
(l)
$dP/dt_{min}$ (mmHg ms$^{-1}$)
-1.5
-1.0
(m)
$dP/dt_{min}$ Rel. Level
0.2
0.4
(n)
$dP/dt_{min}$ Delay (s)
0.078
0.083
(o)
$dP/dt_d$ Delay (s)
0.152
0.157
(p)
$dP/dt_d$ - $dP/dt_{max}$ (mmHg)
-12
-8
(q)
$dP/dt_d$ - $dP/dt_{min}$ (mmHg)
-4
-2
(r)
-25
-20
-15
-10
DiN - AnN (mmHg)

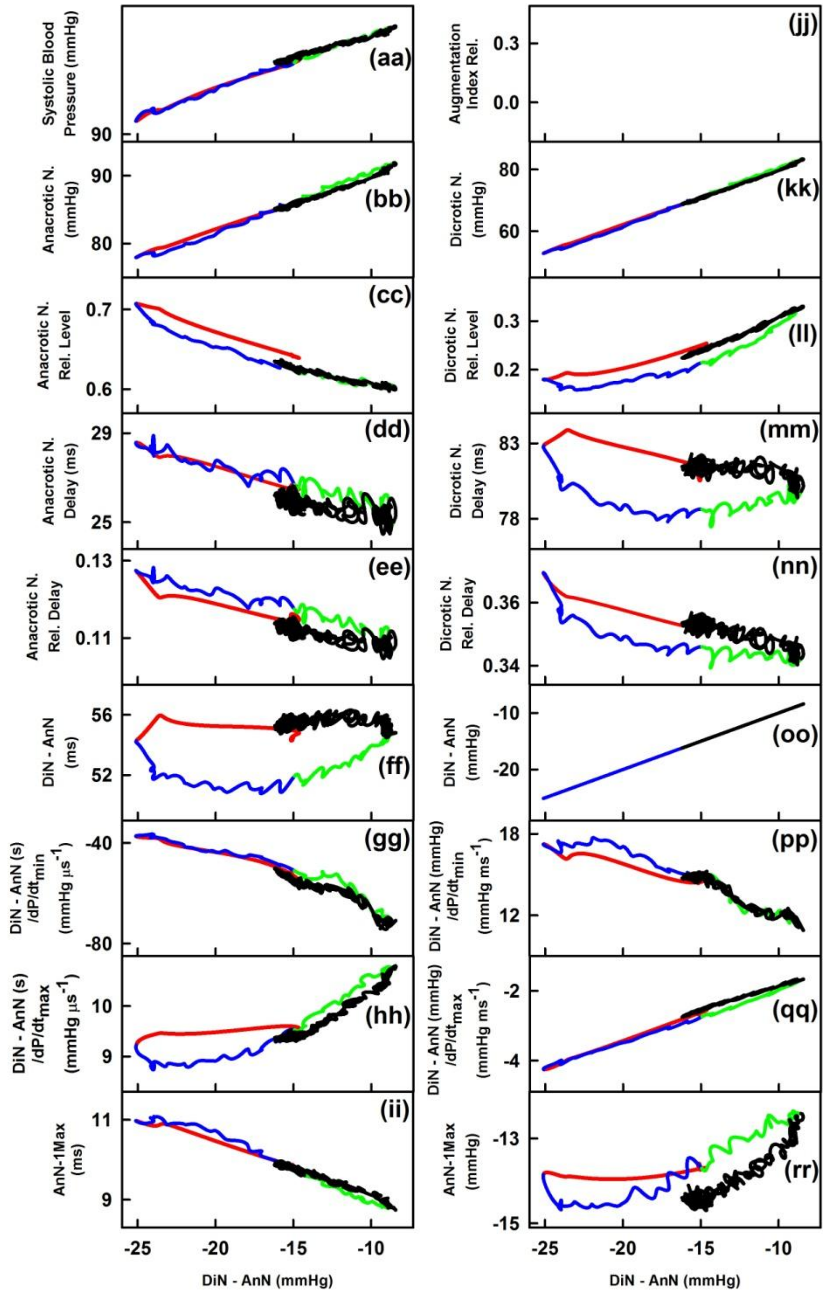


**Figure S2C.** Relationships of HPs to the BP interval between dicrotic (DiN) and anacrotic (AnN) notches after the administration of 32 nmol kg$^{-1}$ GSNO. The colors and time dependent data correspond to Figure 2. The hysteresis was arbitrary defined as HPs-(DiN-AnN in mmHg) loop > 5 mmHg of DiN-AnN. The non-hysteresis was arbitrary defined as HPs-(DiN-AnN in mmHg) loop ≤ 5 mmHg of DiN-AnN.

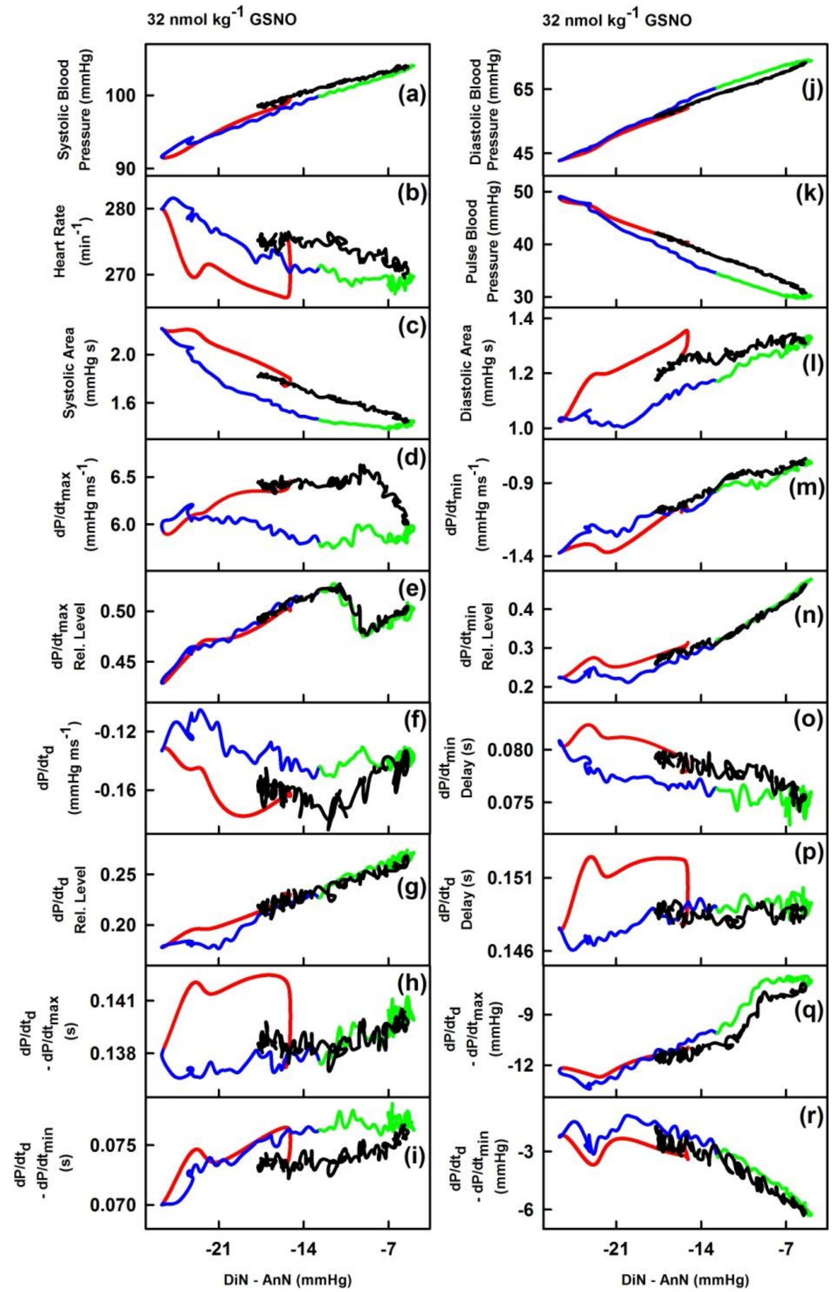

32 nmol kg$^{-1}$ GSNO
32 nmol kg$^{-1}$ GSNO
Systolic Blood Pressure (mmHg)
Heart Rate (min$^{-1}$)
Systolic Area (mmHg s)
$dP/dt_{max}$ (mmHg ms$^{-1}$)
$dP/dt_{max}$ Rel. Level
$dP/dt_d$ (mmHg ms$^{-1}$)
$dP/dt_d$ Rel. Level
$dP/dt_d$ - $dP/dt_{max}$ (s)
$dP/dt_d$ - $dP/dt_{min}$ (s)
Diastolic Blood Pressure (mmHg)
Pulse Blood Pressure (mmHg)
Diastolic Area (mmHg s)
$dP/dt_{min}$ (mmHg ms$^{-1}$)
$dP/dt_{min}$ Rel. Level
$dP/dt_{min}$ Delay (s)
$dP/dt_d$ Delay (s)
$dP/dt_d$ - $dP/dt_{max}$ (mmHg)
$dP/dt_d$ - $dP/dt_{min}$ (mmHg)
(a)
(b)
(c)
(d)
(e)
(f)
(g)
(h)
(i)
(j)
(k)
(l)
(m)
(n)
(o)
(p)
(q)
(r)
DiN - AnN (mmHg)
DiN - AnN (mmHg)

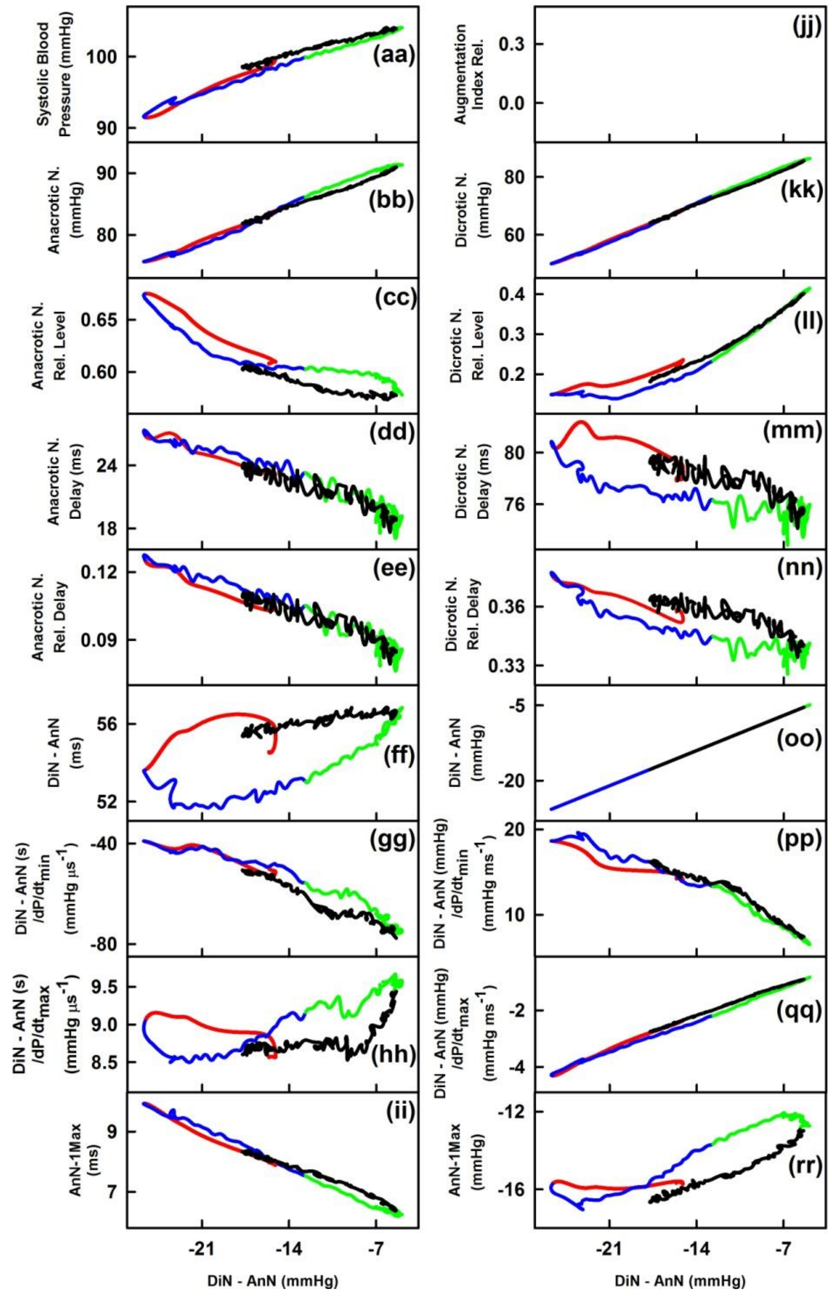


**Figure S2D.** Relationships of HPs to the BP interval between dicrotic (DiN) and anacrotic (AnN) notches after the administration of 32 nmol kg$^{-1}$ GSNO. The colors and time dependent data correspond to Figure 2. The hysteresis was arbitrary defined as HPs-(DiN-AnN in mmHg) loop > 5 mmHg of DiN-AnN. The non-hysteresis was arbitrary defined as HPs-(DiN-AnN in mmHg) loop ≤ 5 mmHg of DiN-AnN.

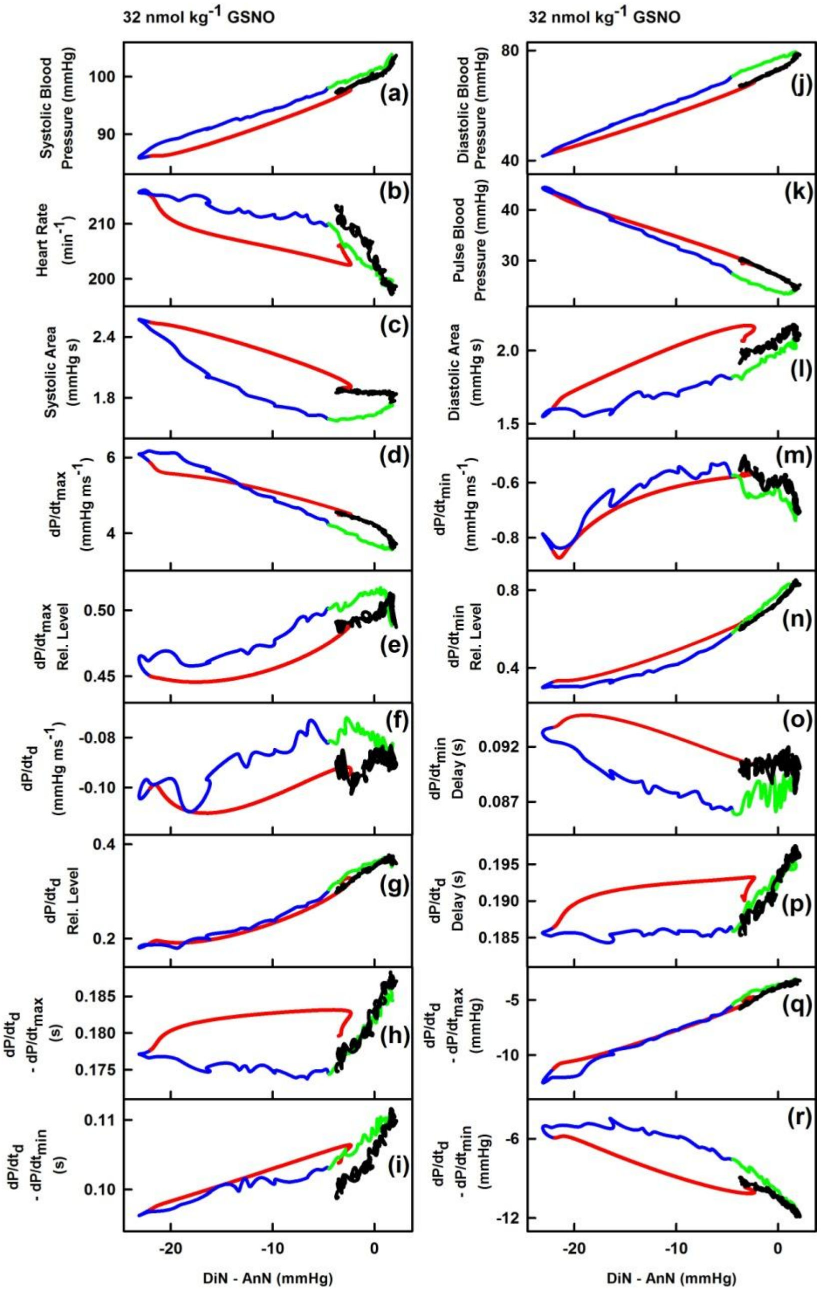

32 nmol kg-1 GSNO
32 nmol kg-1 GSNO
Systolic Blood Pressure (mmHg)
Heart Rate (min-1)
Systolic Area (mmHg s)
dP/dtmax (mmHg ms-1)
dP/dtmax Rel. Level
dP/dtd (mmHg ms-1)
dP/dtd Rel. Level
dP/dtd - dP/dtmax (s)
dP/dtd - dP/dtmin (s)
Diastolic Blood Pressure (mmHg)
Pulse Blood Pressure (mmHg)
Diastolic Area (mmHg s)
dP/dtmin (mmHg ms-1)
dP/dtmin Rel. Level
dP/dtmin Delay (s)
dP/dtd Delay (s)
dP/dtd - dP/dtmax (mmHg)
dP/dtd - dP/dtmin (mmHg)
(a)
(b)
(c)
(d)
(e)
(f)
(g)
(h)
(i)
(j)
(k)
(l)
(m)
(n)
(o)
(p)
(q)
(r)
DiN - AnN (mmHg)
DiN - AnN (mmHg)

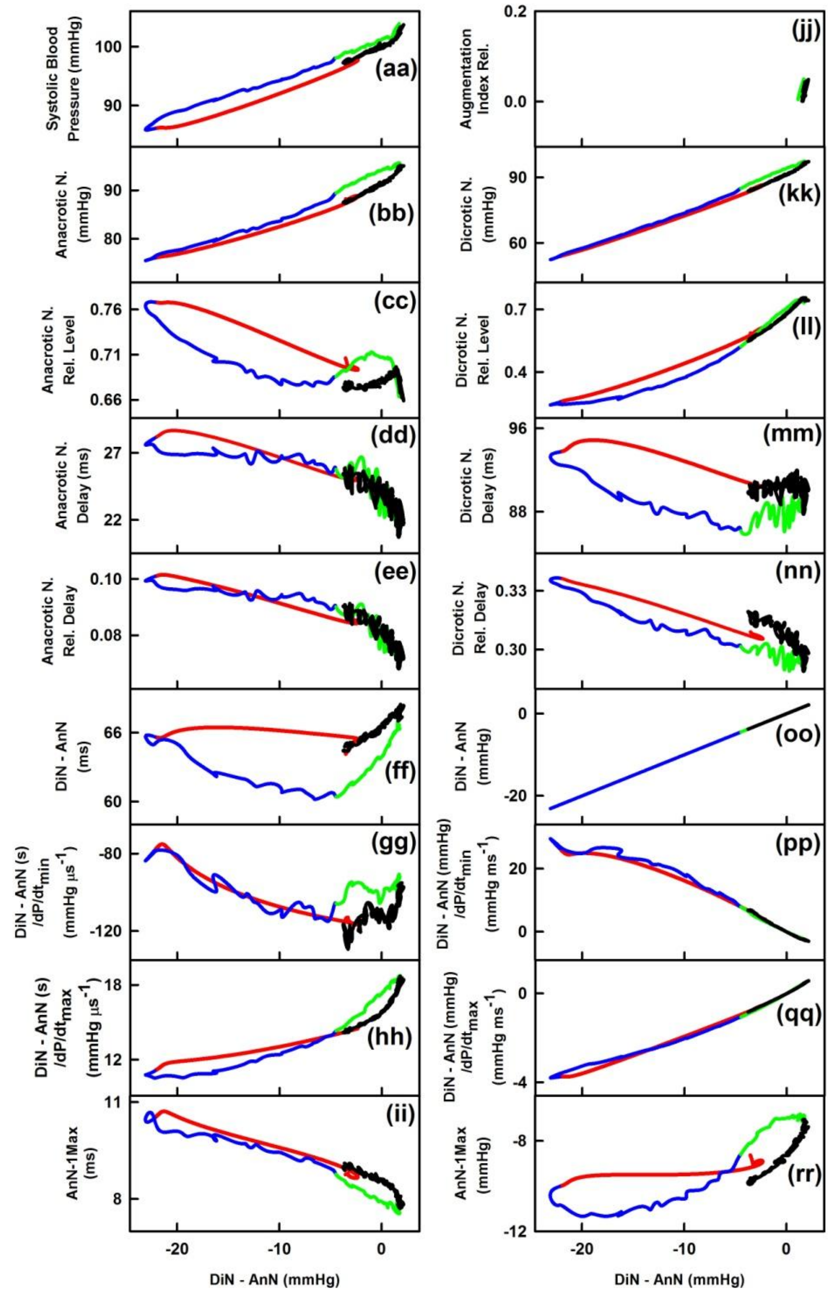


**Figure S2E.** Relationships of HPs to the BP interval between dicrotic (DiN) and anacrotic (AnN) notches after the administration of 32 nmol kg$^{-1}$ GSNO. The colors and time dependent data correspond to Figure 2. The hysteresis was arbitrary defined as HPs-(DiN-AnN in mmHg) loop > 5 mmHg of DiN-AnN. The non-hysteresis was arbitrary defined as HPs-(DiN-AnN in mmHg) loop ≤ 5 mmHg of DiN-AnN.

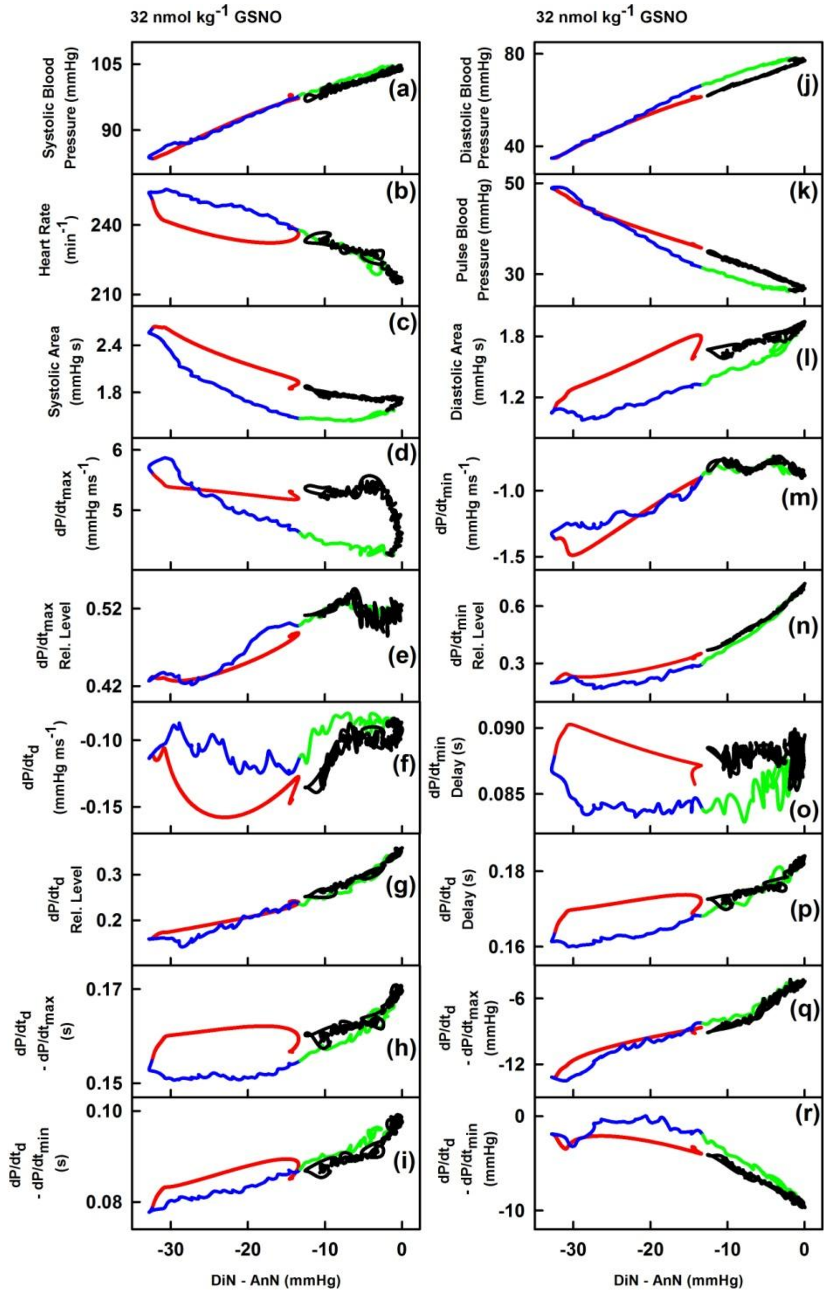

32 nmol kg-1 GSNO
32 nmol kg-1 GSNO
Systolic Blood Pressure (mmHg)
105
90
(a)
Heart Rate (min-1)
240
210
(b)
Systolic Area (mmHg s)
2.4
1.8
(c)
dP/dtmax (mmHg ms-1)
6
5
(d)
dP/dtmax Rel. Level
0.52
0.42
(e)
dP/dtd (mmHg ms-1)
-0.10
-0.15
(f)
dP/dtd Rel. Level
0.3
0.2
(g)
dP/dtd - dP/dtmax (s)
0.17
0.15
(h)
dP/dtd - dP/dtmin (s)
0.10
0.08
(i)
-30
-20
-10
0
DiN - AnN (mmHg)
Diastolic Blood Pressure (mmHg)
80
40
(j)
Pulse Blood Pressure (mmHg)
50
30
(k)
Diastolic Area (mmHg s)
1.8
1.2
(l)
dP/dtmin (mmHg ms-1)
-1.0
-1.5
(m)
dP/dtmin Rel. Level
0.6
0.3
(n)
dP/dtmin Delay (s)
0.090
0.085
(o)
dP/dtd Delay (s)
0.18
0.16
(p)
dP/dtd - dP/dtmax (mmHg)
-6
-12
(q)
dP/dtd - dP/dtmin (mmHg)
0
-10
(r)
-30
-20
-10
0
DiN - AnN (mmHg)

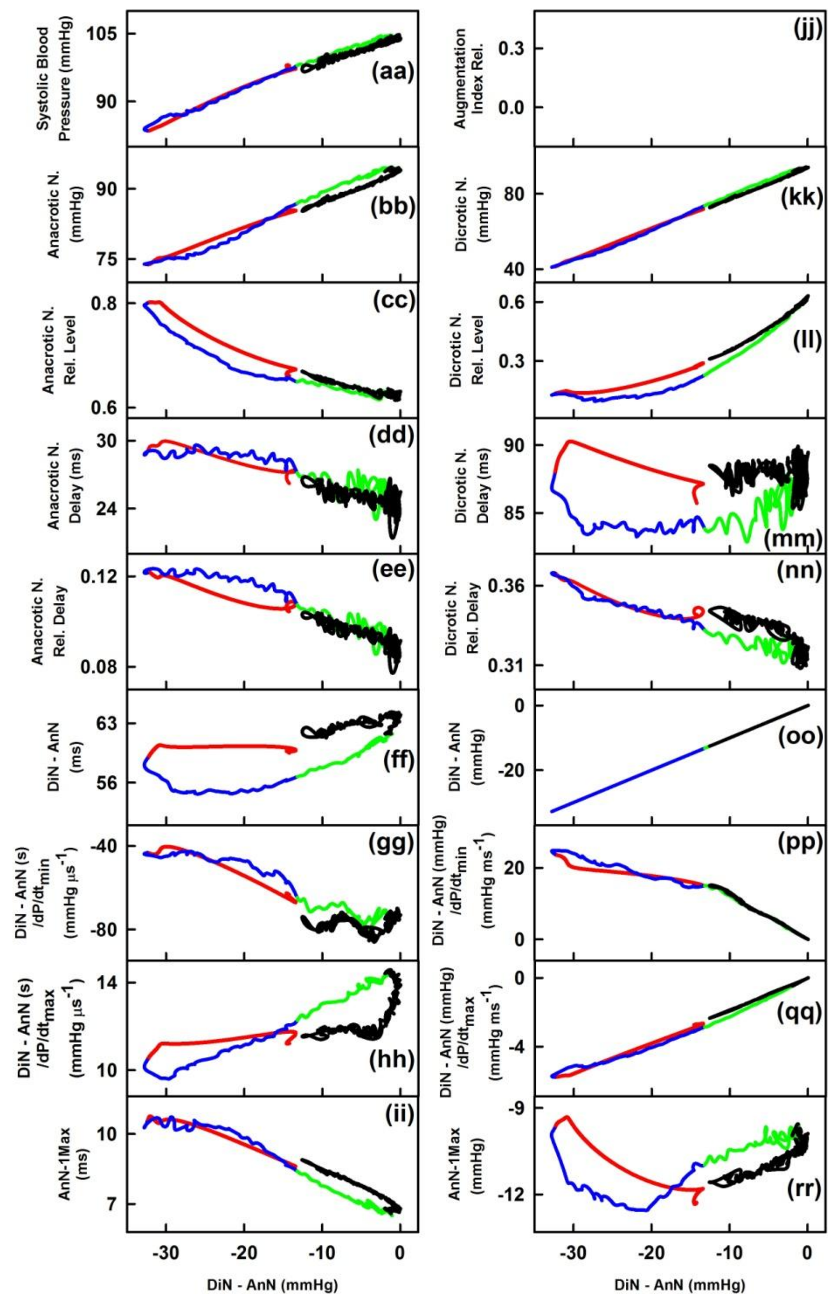


**Figure S2F.** Relationships of HPs to the BP interval between dicrotic (DiN) and anacrotic (AnN) notches after the administration of 32 nmol kg$^{-1}$ GSNO. The colors and time dependent data correspond to Figure 2. The hysteresis was arbitrary defined as HPs-(DiN-AnN in mmHg) loop > 5 mmHg of DiN-AnN. The non-hysteresis was arbitrary defined as HPs-(DiN-AnN in mmHg) loop ≤ 5 mmHg of DiN-AnN.

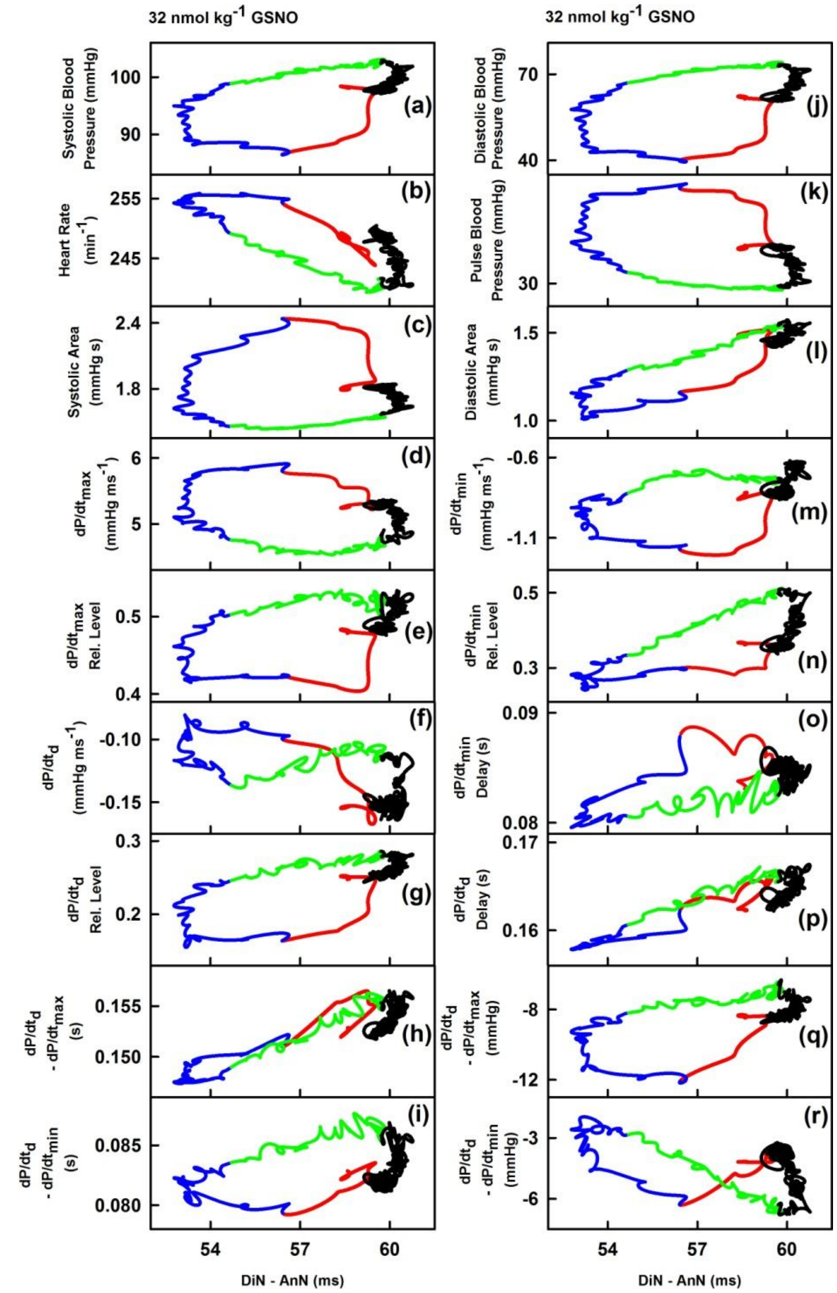
32 nmol kg-1 GSNO
32 nmol kg-1 GSNO
Systolic Blood Pressure (mmHg)
Heart Rate (min-1)
Systolic Area (mmHg s)
dP/dtmax (mmHg ms-1)
dP/dtmax Rel. Level
dP/dtd (mmHg ms-1)
dP/dtd Rel. Level
dP/dtd - dP/dtmax (s)
dP/dtd - dP/dtmin (s)
Diastolic Blood Pressure (mmHg)
Pulse Blood Pressure (mmHg)
Diastolic Area (mmHg s)
dP/dtmin (mmHg ms-1)
dP/dtmin Rel. Level
dP/dtmin Delay (s)
dP/dtd Delay (s)
dP/dtd - dP/dtmax (mmHg)
dP/dtd - dP/dtmin (mmHg)
(a)
(b)
(c)
(d)
(e)
(f)
(g)
(h)
(i)
(j)
(k)
(l)
(m)
(n)
(o)
(p)
(q)
(r)
DiN - AnN (ms)
DiN - AnN (ms)

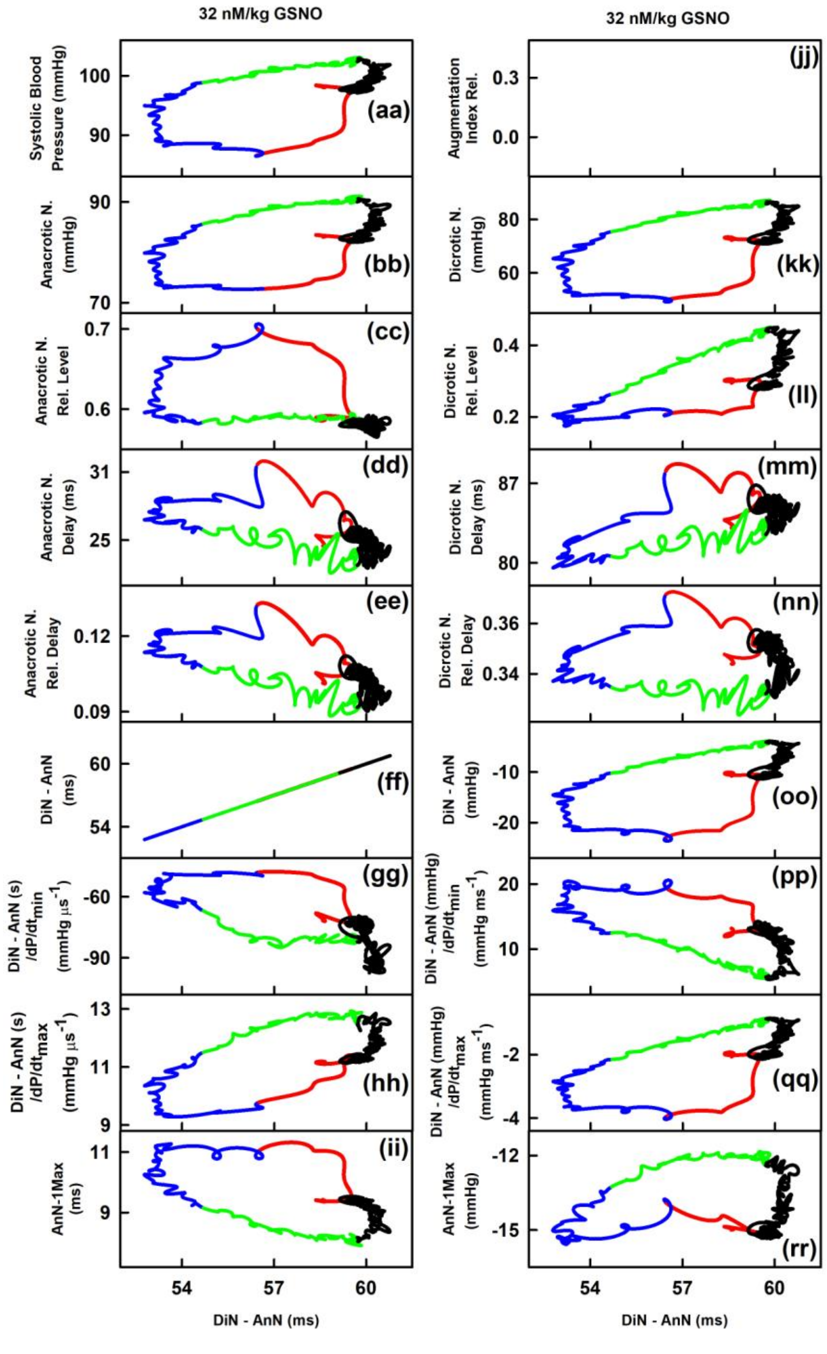


**Figure S3A.** Relationships of HPs to the time interval between dicrotic (DiN) and anacrotic (AnN) notches after the administration of 32 nmol kg$^{-1}$ GSNO. The colors and time dependent data correspond to Figure 2. The hysteresis was arbitrary defined as HPs-(DiN-AnN in ms) loop > 3 ms of DiN-AnN.

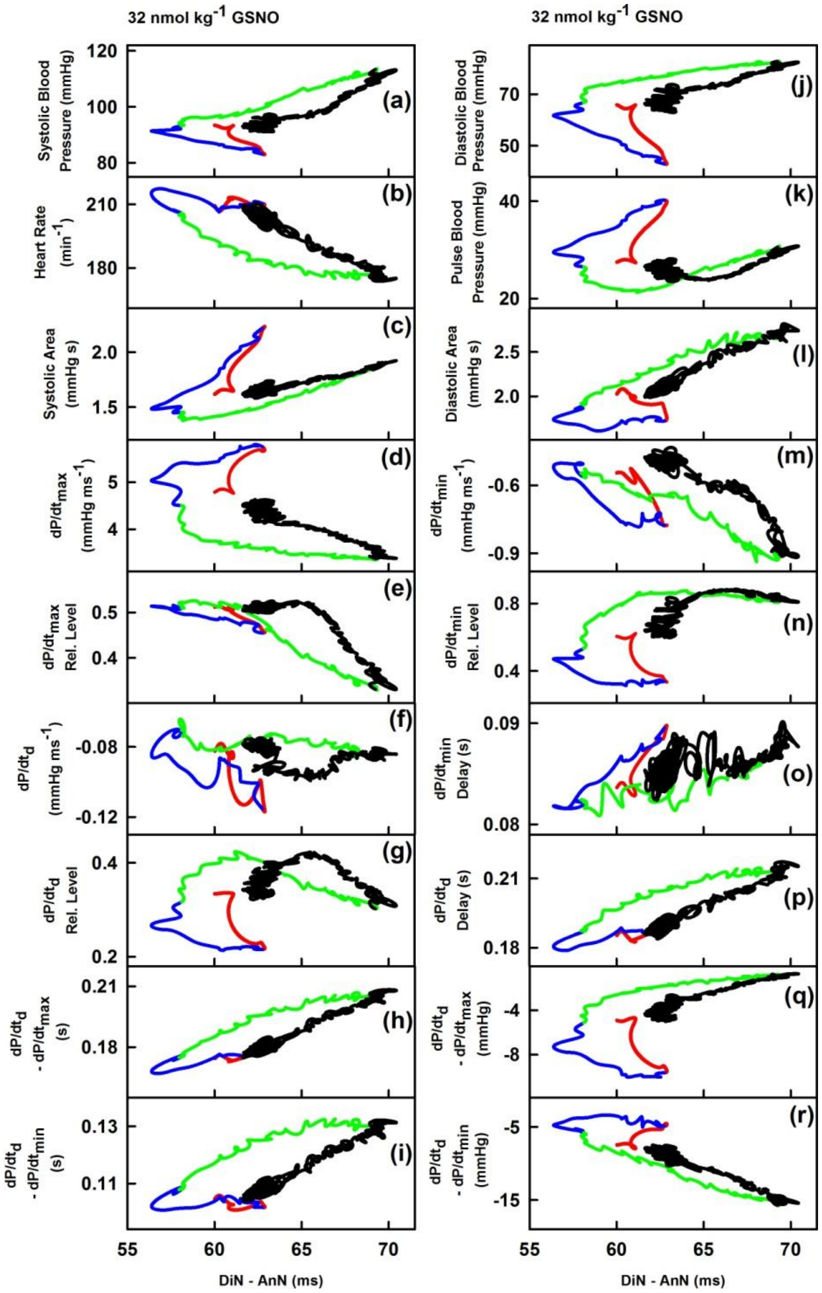

32 nmol kg$^{-1}$ GSNO
32 nmol kg$^{-1}$ GSNO
Systolic Blood Pressure (mmHg)
Heart Rate (min$^{-1}$)
Systolic Area (mmHg s)
$dP/dt_{max}$ (mmHg ms$^{-1}$)
$dP/dt_{max}$ Rel. Level
$dP/dt_d$ (mmHg ms$^{-1}$)
$dP/dt_d$ Rel. Level
$dP/dt_d$ - $dP/dt_{max}$ (s)
$dP/dt_d$ - $dP/dt_{min}$ (s)
Diastolic Blood Pressure (mmHg)
Pulse Blood Pressure (mmHg)
Diastolic Area (mmHg s)
$dP/dt_{min}$ (mmHg ms$^{-1}$)
$dP/dt_{min}$ Rel. Level
$dP/dt_{min}$ Delay (s)
$dP/dt_d$ Delay (s)
$dP/dt_d$ - $dP/dt_{max}$ (mmHg)
$dP/dt_d$ - $dP/dt_{min}$ (mmHg)
(a)
(b)
(c)
(d)
(e)
(f)
(g)
(h)
(i)
(j)
(k)
(l)
(m)
(n)
(o)
(p)
(q)
(r)
DiN - AnN (ms)
DiN - AnN (ms)

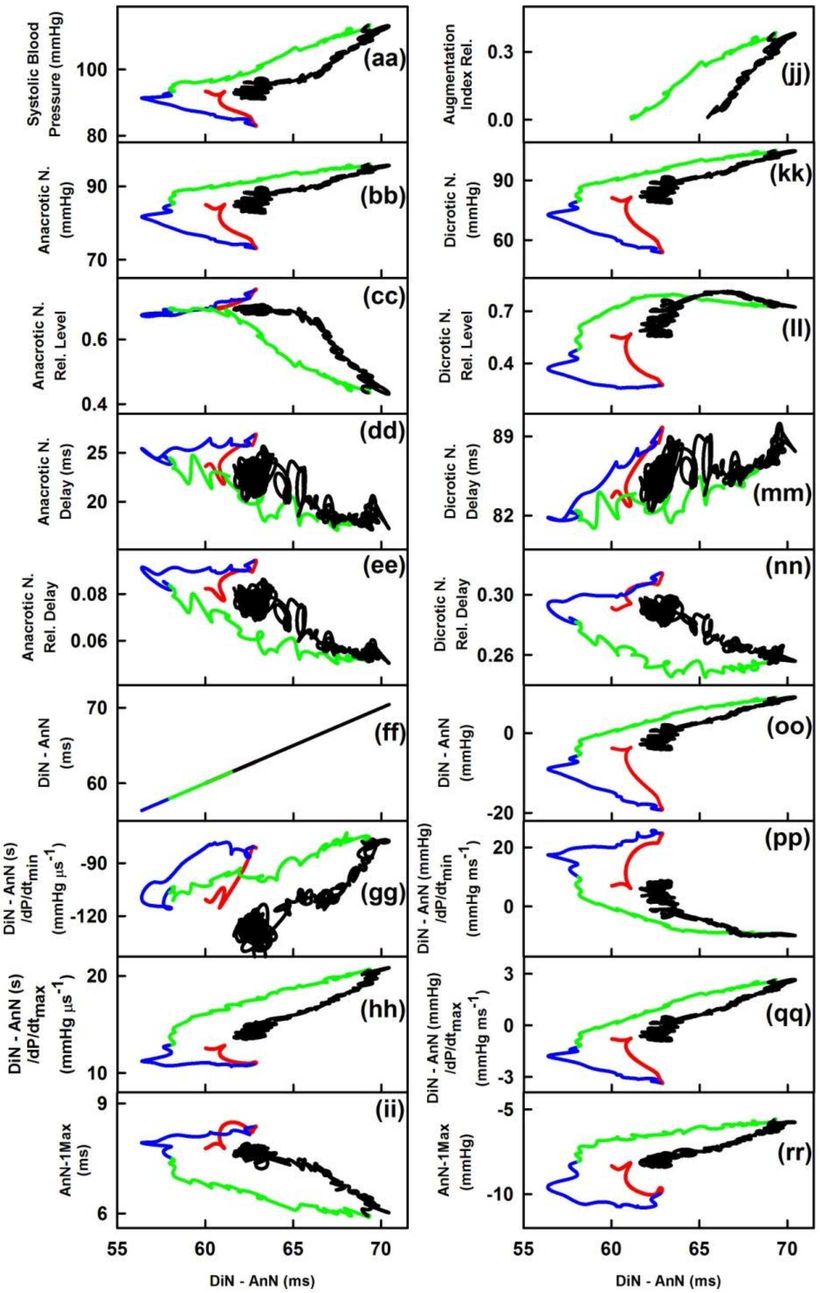


**Figure S3B.** Relationships of HPs to the time interval between dicrotic (DiN) and anacrotic (AnN) notches after the administration of 32 nmol kg$^{-1}$ GSNO. The colors and time dependent data correspond to Figure 2. The hysteresis was arbitrary defined as HPs-(DiN-AnN in ms) loop > 3 ms of DiN-AnN.

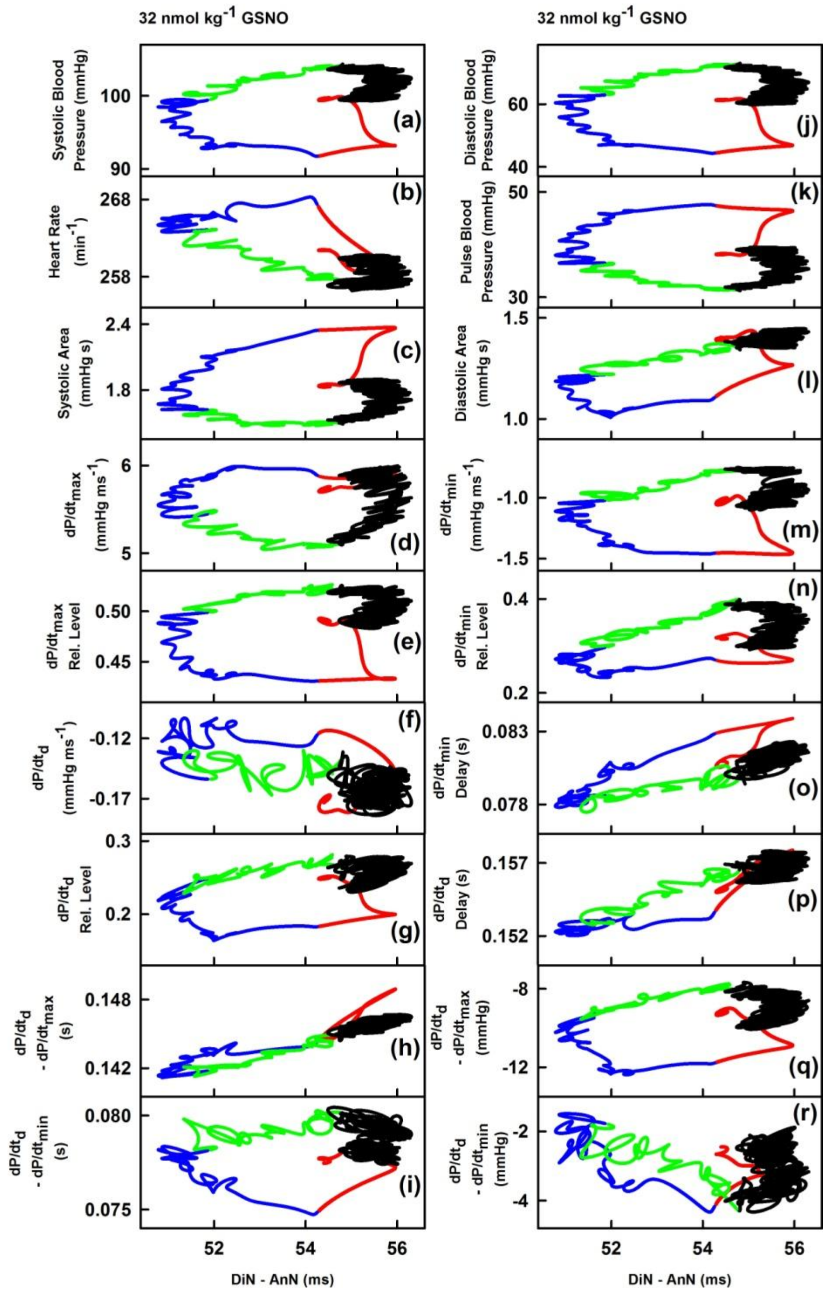

32 nmol kg-1 GSNO
32 nmol kg-1 GSNO
Systolic Blood Pressure (mmHg)
Heart Rate (min-1)
Systolic Area (mmHg s)
dP/dtmax (mmHg ms-1)
dP/dtmax Rel. Level
dP/dtd (mmHg ms-1)
dP/dtd Rel. Level
dP/dtd - dP/dtmax (s)
dP/dtd - dP/dtmin (s)
Diastolic Blood Pressure (mmHg)
Pulse Blood Pressure (mmHg)
Diastolic Area (mmHg s)
dP/dtmin (mmHg ms-1)
dP/dtmin Rel. Level
dP/dtmin Delay (s)
dP/dtd Delay (s)
dP/dtd - dP/dtmax (mmHg)
dP/dtd - dP/dtmin (mmHg)
(a)
(b)
(c)
(d)
(e)
(f)
(g)
(h)
(i)
(j)
(k)
(l)
(m)
(n)
(o)
(p)
(q)
(r)
DiN - AnN (ms)
DiN - AnN (ms)

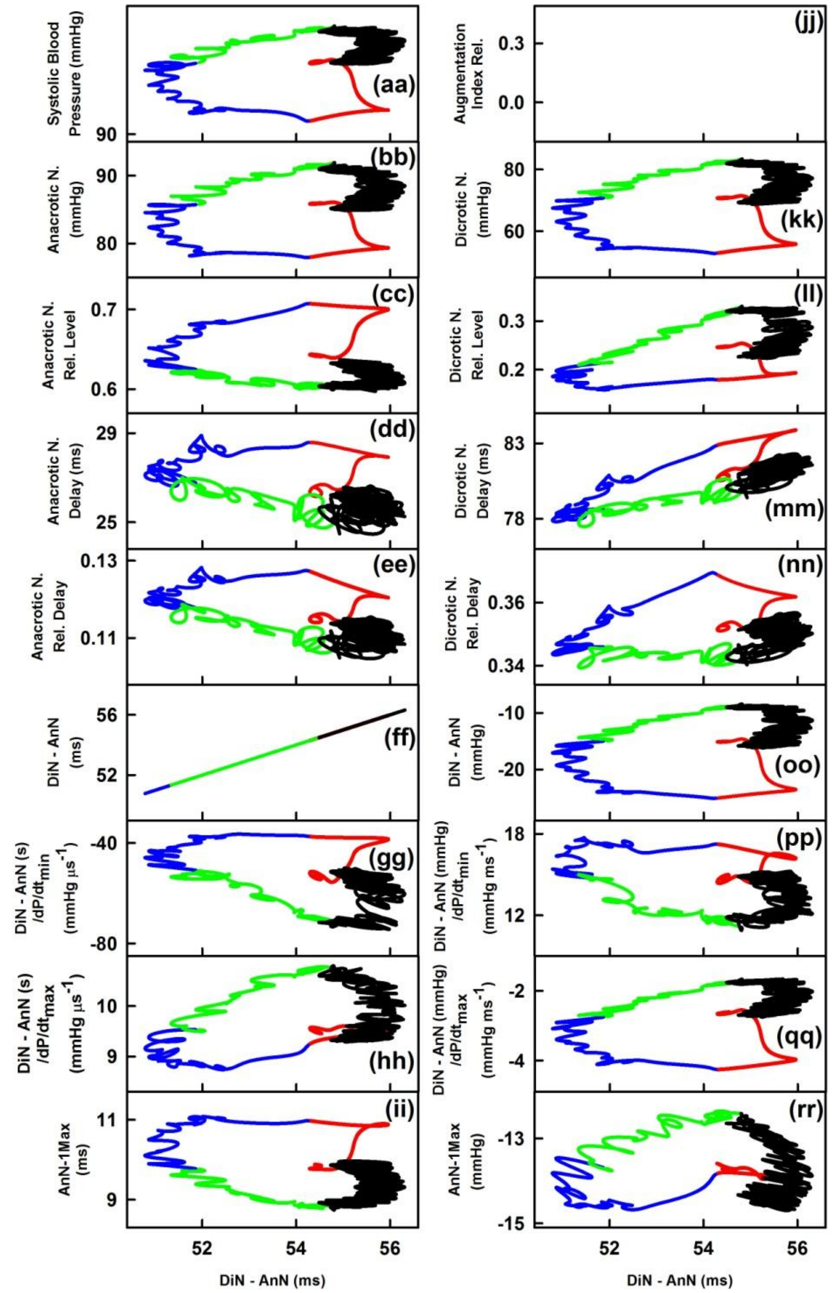


**Figure S3C.** Relationships of HPs to the time interval between dicrotic (DiN) and anacrotic (AnN) notches after the administration of 32 nmol kg$^{-1}$ GSNO. The colors and time dependent data correspond to Figure 2. The hysteresis was arbitrary defined as HPs-(DiN-AnN in ms) loop > 3 ms of DiN-AnN.

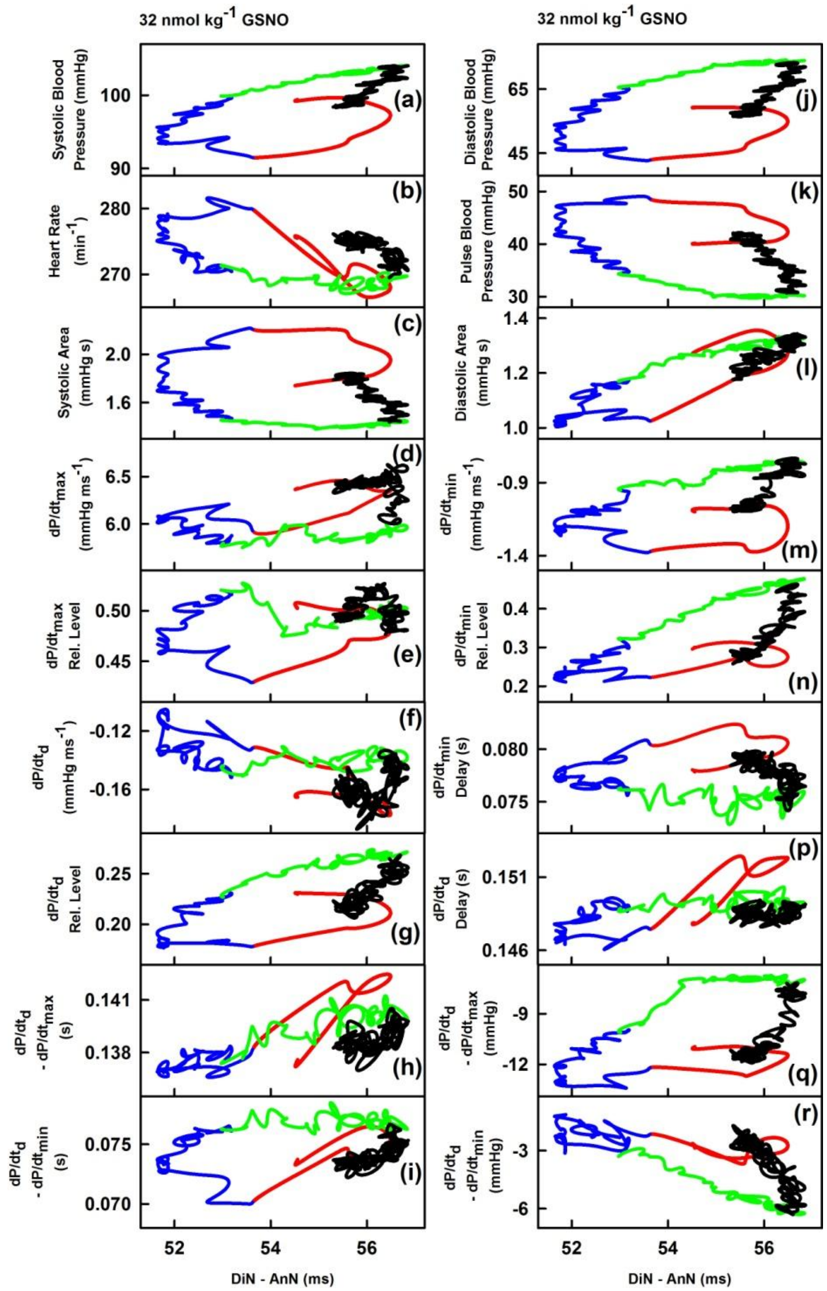
32 nmol kg-1 GSNO
32 nmol kg-1 GSNO
Systolic Blood Pressure (mmHg)
Heart Rate (min-1)
Systolic Area (mmHg s)
dP/dtmax (mmHg ms-1)
dP/dtmax Rel. Level
dP/dtd (mmHg ms-1)
dP/dtd Rel. Level
dP/dtd - dP/dtmax (s)
dP/dtd - dP/dtmin (s)
Diastolic Blood Pressure (mmHg)
Pulse Blood Pressure (mmHg)
Diastolic Area (mmHg s)
dP/dtmin (mmHg ms-1)
dP/dtmin Rel. Level
dP/dtmin Delay (s)
dP/dtd Delay (s)
dP/dtd - dP/dtmax (mmHg)
dP/dtd - dP/dtmin (mmHg)
(a)
(b)
(c)
(d)
(e)
(f)
(g)
(h)
(i)
(j)
(k)
(l)
(m)
(n)
(o)
(p)
(q)
(r)
DiN - AnN (ms)
DiN - AnN (ms)

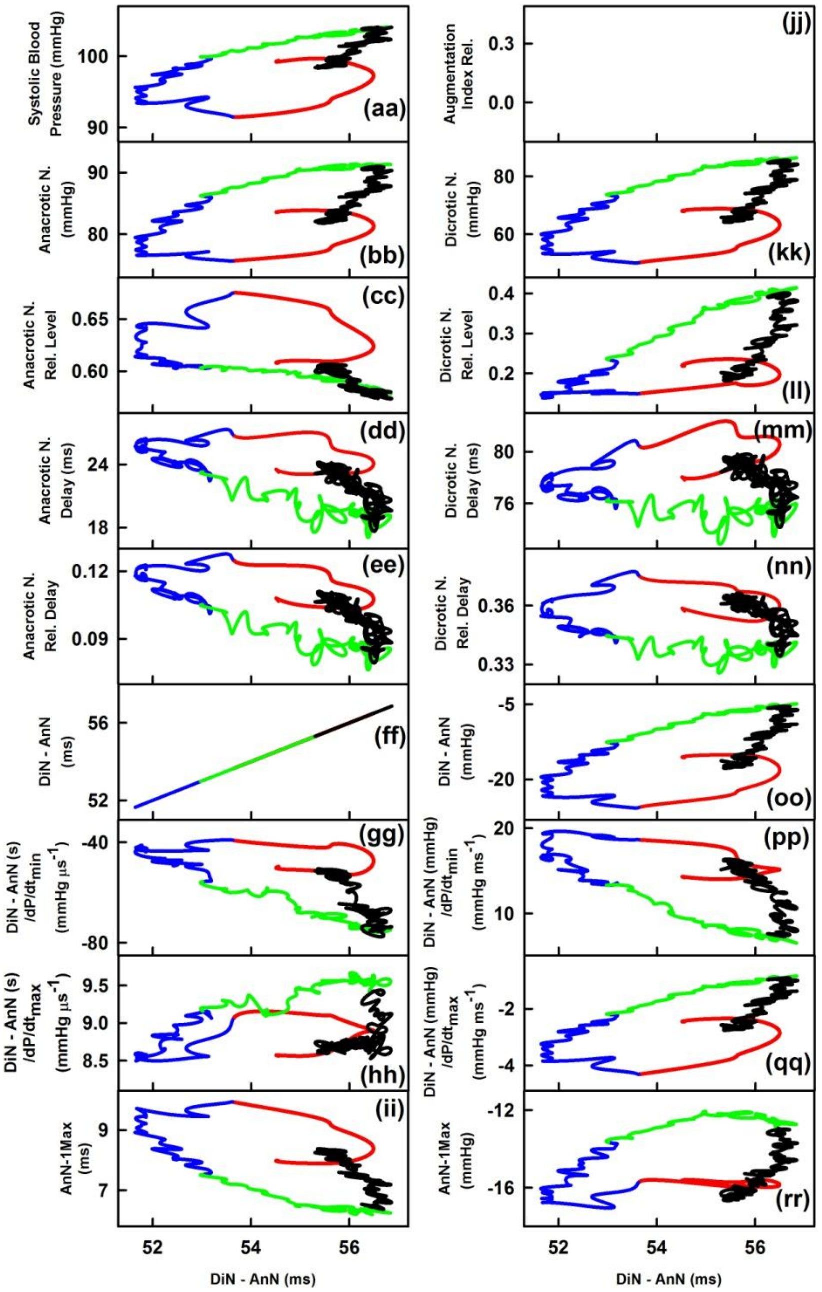


**Figure S3D.** Relationships of HPs to the time interval between dicrotic (DiN) and anacrotic (AnN) notches after the administration of 32 nmol kg$^{-1}$ GSNO. The colors and time dependent data correspond to Figure 2. The hysteresis was arbitrary defined as HPs-(DiN-AnN in ms) loop > 3 ms of DiN-AnN.

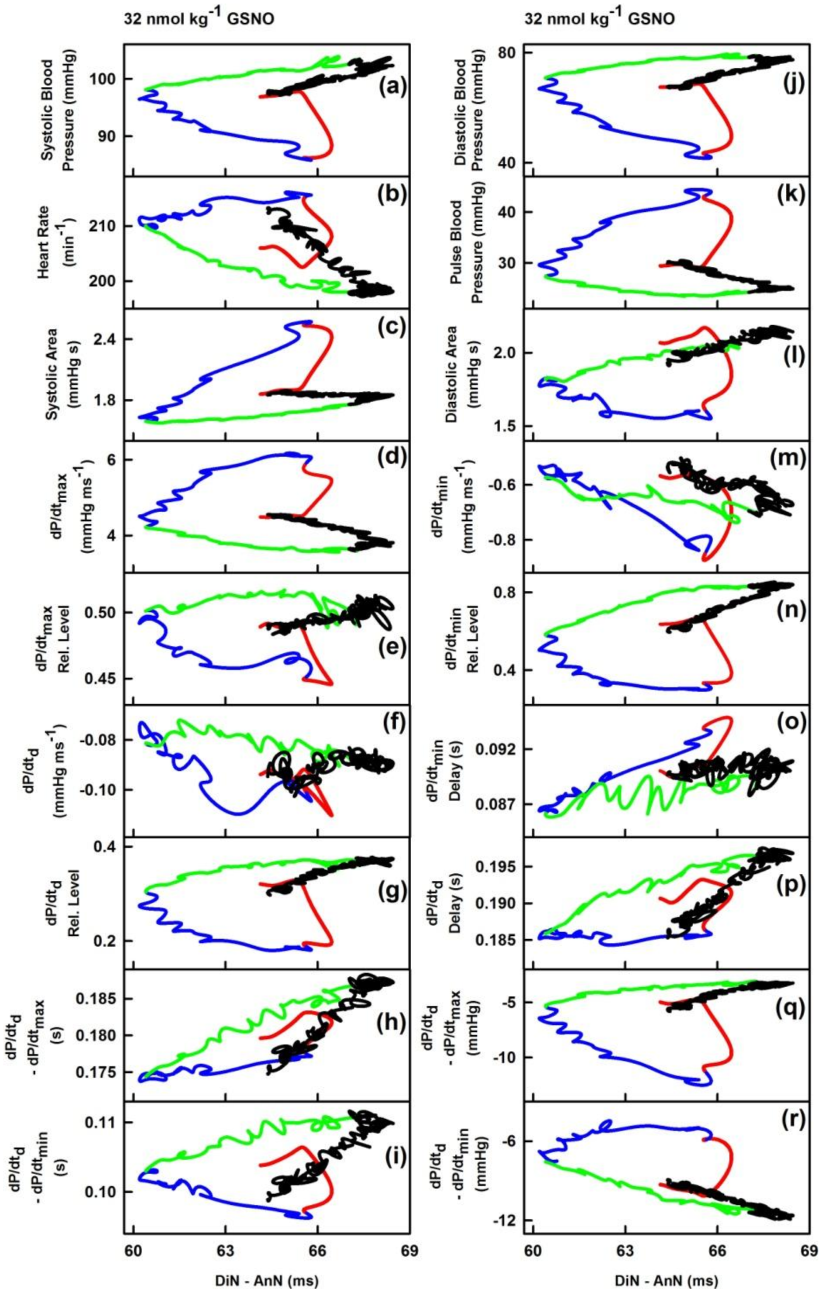

32 nmol kg$^{-1}$ GSNO
32 nmol kg$^{-1}$ GSNO
Systolic Blood Pressure (mmHg)
Heart Rate (min$^{-1}$)
Systolic Area (mmHg s)
$dP/dt_{max}$ (mmHg ms$^{-1}$)
$dP/dt_{max}$ Rel. Level
$dP/dt_d$ (mmHg ms$^{-1}$)
$dP/dt_d$ Rel. Level
$dP/dt_d$ - $dP/dt_{max}$ (s)
$dP/dt_d$ - $dP/dt_{min}$ (s)
Diastolic Blood Pressure (mmHg)
Pulse Blood Pressure (mmHg)
Diastolic Area (mmHg s)
$dP/dt_{min}$ (mmHg ms$^{-1}$)
$dP/dt_{min}$ Rel. Level
$dP/dt_{min}$ Delay (s)
$dP/dt_d$ Delay (s)
$dP/dt_d$ - $dP/dt_{max}$ (mmHg)
$dP/dt_d$ - $dP/dt_{min}$ (mmHg)
(a)
(b)
(c)
(d)
(e)
(f)
(g)
(h)
(i)
(j)
(k)
(l)
(m)
(n)
(o)
(p)
(q)
(r)
DiN - AnN (ms)
DiN - AnN (ms)

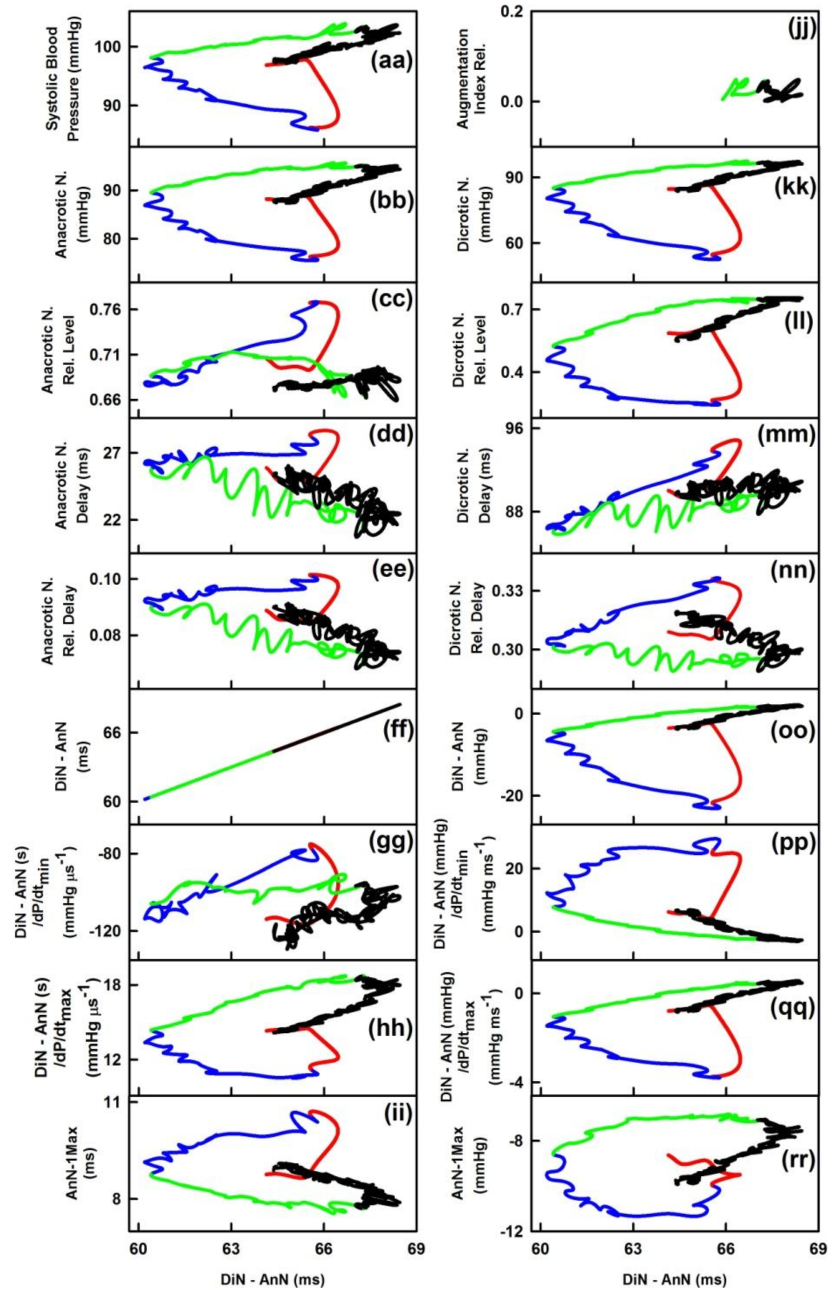


**Figure S3E.** Relationships of HPs to the time interval between dicrotic (DiN) and anacrotic (AnN) notches after the administration of 32 nmol $kg^{-1}$ GSNO. The colors and time dependent data correspond to Figure 2. The hysteresis was arbitrary defined as HPs-(DiN-AnN in ms) loop > 3 ms of DiN-AnN.

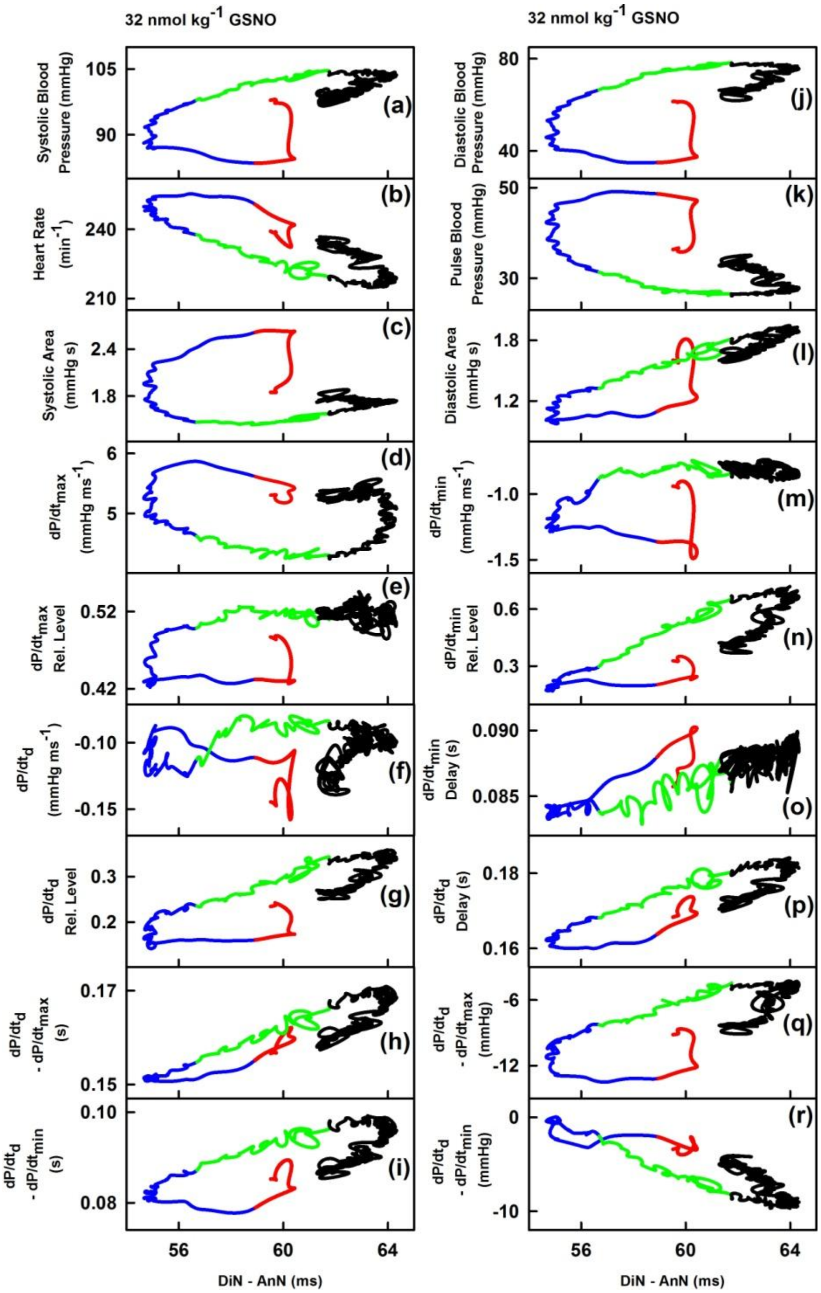

32 nmol kg-1 GSNO
32 nmol kg-1 GSNO
Systolic Blood Pressure (mmHg)
Heart Rate (min-1)
Systolic Area (mmHg s)
dP/dtmax (mmHg ms-1)
dP/dtmax Rel. Level
dP/dtd (mmHg ms-1)
dP/dtd Rel. Level
dP/dtd - dP/dtmax (s)
dP/dtd - dP/dtmin (s)
Diastolic Blood Pressure (mmHg)
Pulse Blood Pressure (mmHg)
Diastolic Area (mmHg s)
dP/dtmin (mmHg ms-1)
dP/dtmin Rel. Level
dP/dtmin Delay (s)
dP/dtd Delay (s)
dP/dtd - dP/dtmax (mmHg)
dP/dtd - dP/dtmin (mmHg)
(a)
(b)
(c)
(d)
(e)
(f)
(g)
(h)
(i)
(j)
(k)
(l)
(m)
(n)
(o)
(p)
(q)
(r)
DiN - AnN (ms)
DiN - AnN (ms)

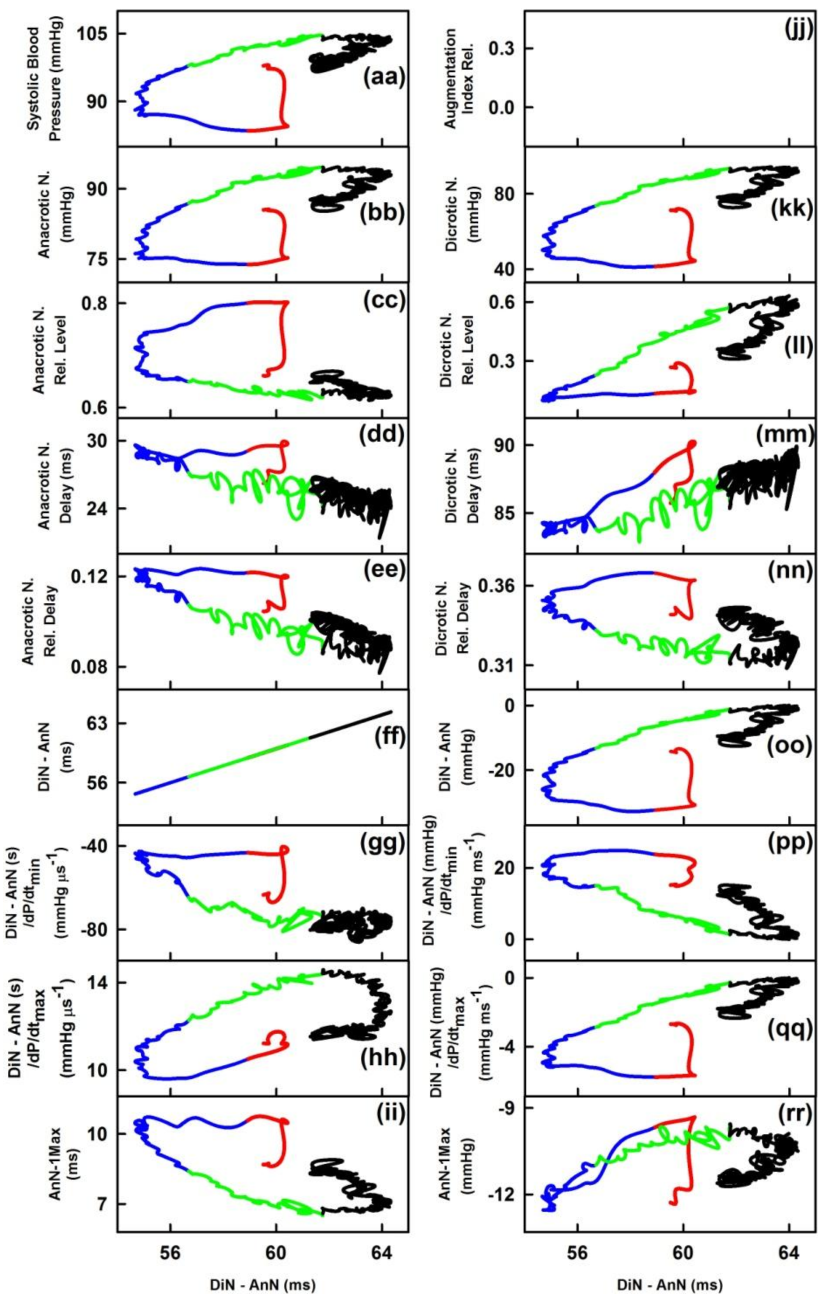


**Figure S3F.** Relationships of HPs to the time interval between dicrotic (DiN) and anacrotic (AnN) notches after the administration of 32 nmol $kg^{-1}$ GSNO. The colors and time dependent data correspond to Figure 2. The hysteresis was arbitrary defined as HPs-(DiN-AnN in ms) loop > 3 ms of DiN-AnN.
